# The Janus face of high trans-effect carbenes in olefin metathesis: gateway to both productivity and decomposition†

**DOI:** 10.1039/d2sc00855f

**Published:** 2022-03-22

**Authors:** Giovanni Occhipinti, Daniel L. Nascimento, Marco Foscato, Deryn E. Fogg, Vidar R. Jensen

**Affiliations:** Department of Chemistry, University of Bergen Allégaten 41 N-5007 Bergen Norway Giovanni.Occhipinti@uib.no Deryn.Fogg@uib.no Vidar.Jensen@uib.no; Center for Catalysis Research & Innovation, Department of Chemistry and Biomolecular Sciences, University of Ottawa Ottawa Canada K1N 6N5 dfogg@uottawa.ca

## Abstract

Ruthenium–cyclic(alkyl)(amino)carbene (CAAC) catalysts, used at ppm levels, can enable dramatically higher productivities in olefin metathesis than their N-heterocyclic carbene (NHC) predecessors. A key reason is the reduced susceptibility of the metallacyclobutane (MCB) intermediate to decomposition *via* β-H elimination. The factors responsible for promoting or inhibiting β-H elimination are explored *via* density functional theory (DFT) calculations, in metathesis of ethylene or styrene (a representative 1-olefin) by Ru–CAAC and Ru–NHC catalysts. Natural bond orbital analysis of the frontier orbitals confirms the greater strength of the orbital interactions for the CAAC species, and the consequent increase in the carbene trans influence and trans effect. The higher trans effect of the CAAC ligands inhibits β-H elimination by destabilizing the transition state (TS) for decomposition, in which an agostic MCB C_β_–H bond is positioned trans to the carbene. Unproductive cycling with ethylene is also curbed, because ethylene is trans to the carbene ligand in the square pyramidal TS for ethylene metathesis. In contrast, metathesis of styrene proceeds *via* a ‘late’ TS with approximately trigonal bipyramidal geometry, in which carbene trans effects are reduced. Importantly, however, the positive impact of a strong trans-effect ligand in limiting β-H elimination is offset by its potent accelerating effect on bimolecular coupling, a major competing means of catalyst decomposition. These two decomposition pathways, known for decades to limit productivity in olefin metathesis, are revealed as distinct, antinomic, responses to a single underlying phenomenon. Reconciling these opposing effects emerges as a clear priority for design of robust, high-performing catalysts.

## Abstract 2

In ruthenium catalysts for olefin metathesis, carbene ligands of high trans influence/effect suppress decomposition *via* β-H elimination, but increase susceptibility to bimolecular decomposition.

## Graphical abstract

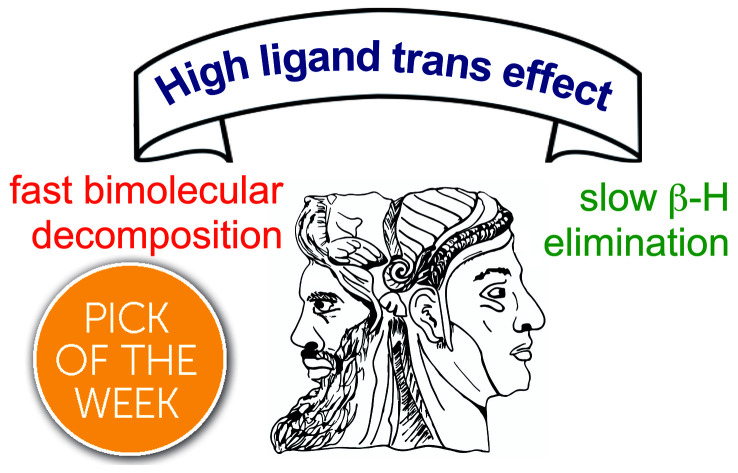

## Introduction

Olefin metathesis is prized for its versatility in enabling the catalytic assembly of unactivated alkenes.^1,2^ Long embraced in organic synthesis, metathesis methodologies are increasingly prominent in frontier applications in materials science^3–19^ and chemical biology,^20–22^ and in hybrid technologies such as DNA-encoded chemical libraries (DECL).^23–25^ Recognized in these and a myriad of other applications (notably pharmaceutical manufacturing)^26–29^ are challenges arising from catalyst decomposition. Indeed, despite a handful of examples in specialty-chemicals and pharmaceutical manufacturing,^26^ industrial uptake of molecular olefin metathesis catalysts has been much slower than anticipated when the ruthenium catalysts were first developed in the 1990s.^30^

**Chart 1:**
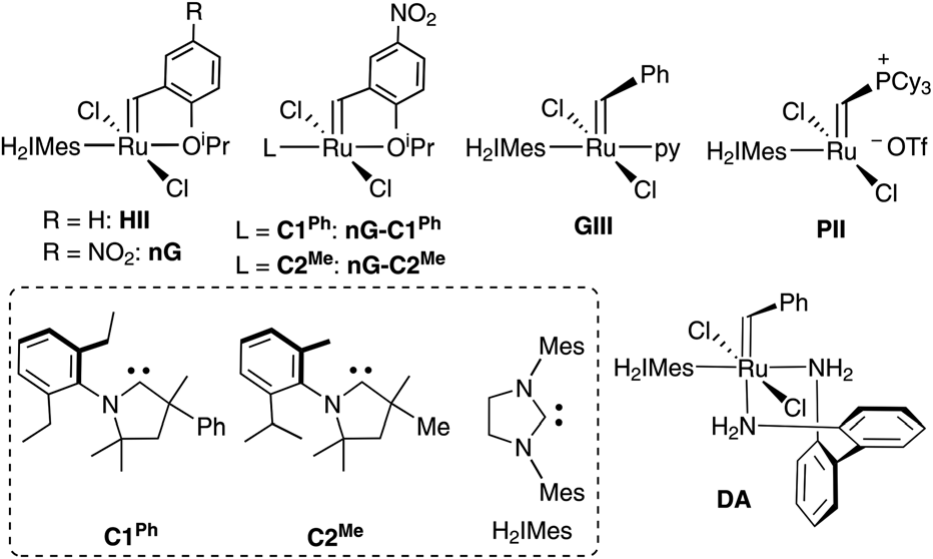
Catalysts and carbene ligands discussed. The CAAC labelling system adopted^42^ (C#^R^) numbers these ligands by common NAr moiety. The superscript R specifies the variable substituent on the quaternary site α to the carbene carbon.

Much effort has been committed to identifying the pathways that underlie decomposition of the widely-used “second-generation” ruthenium catalysts^31–33^ (in particular, the Hoveyda and nitro-Grela catalysts HII and nG; Chart 1, and their PCy_3_-stabilized predecessors, the Grubbs catalysts). Now well established are the mechanisms of degradation by nucleophiles^34–37^ and Brønsted base.^37,38^ As well, in advances critical for applications in chemical biology and related contexts (including DECL technology), we are beginning to understand how these catalysts decompose in water-rich environments.^5,39–41^

**Scheme 1:**
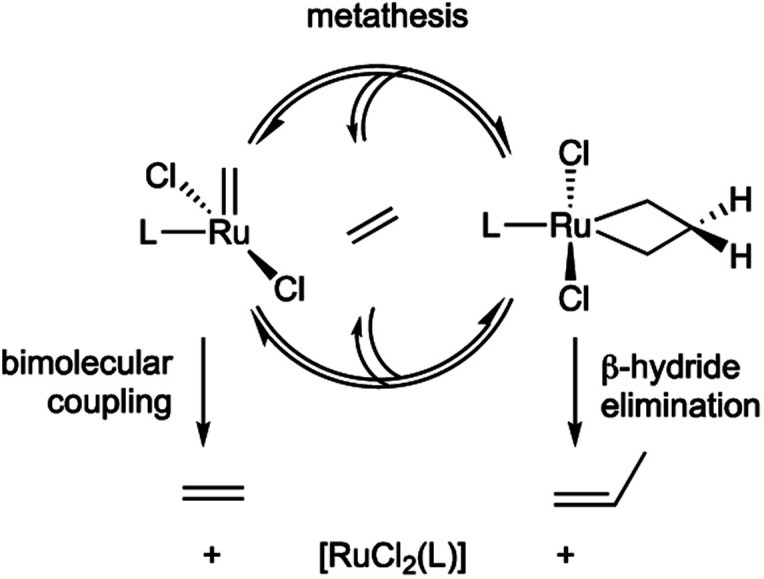
Decomposition of intermediates in Ru-catalyzed olefin metathesis *via* bimolecular coupling or β-H elimination.

The dominant *intrinsic* decomposition modes – that is, pathways inherent to the catalysts themselves – involve bimolecular coupling of the [M]=CH_2_ intermediates, and β-H elimination of the metallacyclobutane (MCB; Scheme 1).^31–33^ We recently reported the first detailed mechanistic insights into the factors that govern bimolecular decomposition.^42,43^ In contrast, the factors that cause β-H elimination of the MCB ring are not discussed even in comprehensive reviews,^31–33^ despite the fact that this pathway has been recognized for decades for both d^0^ catalysts^44^ and the more robust Ru systems.^45,46^

In the broader context, the factors that promote or suppress β-H elimination for a given organometallic complex are incompletely resolved. Textbook requirements^47^ are a vacant site cis to the alkyl ligand, and the capacity to adopt a *syn*-coplanar arrangement of the M–C_α_–C_β_–H moiety (but see below). Coordinative saturation and ligand bulk or rigidity can thus inhibit β-H elimination.^47–50^ Recent studies reinforce the critical role of steric and geometric factors in enabling C–H agostic binding.^49,51^ Aside from the requirement of an empty metal d orbital to accept electron density from the C_β_–H bond (the latter accounting for the known stability of d^10^ metal alkyls),^48,50^ electronic effects are less clear-cut.^47–59^ Whereas a more electron-rich metal has been suggested to accelerate C_β_–H bond-breaking,^54^ high trans-influence^60^ ligands (typically strong donors, which increase electron density at the metal) have been reported to inhibit β-H elimination by destabilizing the required transition-state species.^52,53,57–59^

Insights into the parameters that govern β-H elimination have broad relevance in catalysis, given the central, enabling role of β-H elimination in certain contexts (*e.g.*, the Shell Higher Olefin Process,^61^ Mizoroki–Heck coupling),^62,63^ and its detrimental role in others (*e.g.*, olefin polymerization,^64^ Suzuki–Miyaura coupling,^65^ ring-closing or cross-metathesis (RCM, CM) of terminal olefins). Critical in the latter two reactions is formation of an unsubstituted MCB that is particularly susceptible to β-H elimination.^66^ This vulnerability underlies the highly detrimental impact of ethylene on metathesis by Ru–NHC and Ru–phosphine catalysts,^67^ documented in process chemistry,^26–29^ continuous-flow metathesis,^68–72^ and in the renewables sector,^26,73,74^ where CM with ethylene (‘ethenolysis’) would otherwise offer the simplest, most powerful means of transforming fatty acid methyl esters (FAMEs) into α-olefins. Less discussed, but likewise critical, are the implications for stereoselective olefin metathesis, given the ability of isomerization-active catalyst decomposition products to erode the selectivity designed into the precatalysts.^75–77^

Until very recently, the ethylene-sensitivity of the ruthenium catalysts, and their susceptibility to decomposition *via* β-H elimination, have resisted solution. Because the latter reaction is unimolecular, it cannot be addressed by catalyst immobilization or use of high-dilution conditions. A lack of consensus on the factors responsible has hampered efforts to achieve highly productive Ru catalysts *via* rational catalyst redesign. The experimental finding that Ru–CAAC catalysts resist β-H elimination,^78^ unlike their first- and second-generation Ru–phosphine and Ru–NHC predecessors, is thus important. In practical terms, this stability contributes to the unprecedented productivity reported for CAAC catalysts at ppm loadings in RCM macrocyclization,^79,80^ ethenolysis of FAMEs,^80–82^ and acrylonitrile CM.^79^ More fundamentally, it offers new opportunities to clarify the factors that promote or inhibit β-H elimination.

Clarifying these factors is the main objective of the present work. To that end, we compare Ru–NHC and Ru–CAAC catalysts for which the susceptibility or resistance to β-H elimination, respectively, are established experimentally. We demonstrate that the high trans effect of the CAAC ligand, a consequence of the strength of the Ru–CAAC bond, is responsible for suppressing this decomposition pathway. The capacity of a high trans-effect ligand to inhibit β-H elimination indeed merits much broader recognition than it has received to date. In the context of olefin metathesis, this labilizing effect holds added importance: it is known to have a further, deleterious impact, accelerating decomposition *via* bimolecular coupling of [M]=CH_2_ intermediates. These two decomposition pathways, known for decades to limit productivity in olefin metathesis, are thus seen for the first time to be related: they are opposing responses to the strong trans effect arising from strong metal-carbene binding.

## Results and discussion

### Assessing the proportion of β-H elimination *vs.* bimolecular coupling

**Fig. 1:**
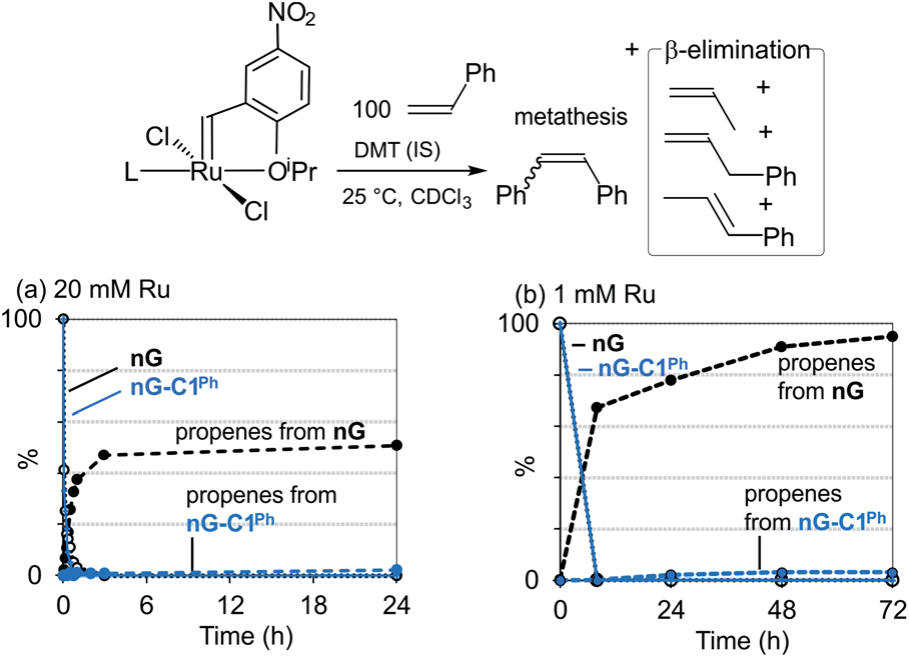
Decomposition of nG and nG-C1^Ph^*via* β-H elimination: disappearance of NMR signals for [Ru] = C*H*Ar, and appearance of signals for propenes. (a) At 20 mM Ru (300 MHz). (b) At 1 mM Ru (500 MHz).

In a prior experimental study, we demonstrated that decomposition of the CAAC catalysts nG-C1^Ph^ and nG-C2^Me^ occurs almost solely *via* bimolecular coupling (BMC).^78^ Contrasting behavior was observed for H_2_IMes catalysts (HII, nG, PII, DA, GIII), all of which decomposed *via* a combination of BMC and β-H elimination, with the exception of GIII.^43^ Diagnostic for β-H elimination is the observation of propene products, formed *via* loss of the metallacyclobutane ring. While the yield of propenes for nG-C1^Ph^ or nG-C2^Me^ was nearly nil, it was >50% for, *e.g.*, the widely-used Grela catalyst nG (Fig. 1a). The H_2_IMes systems clearly decompose *via* competing unimolecular and bimolecular pathways.

Importantly, however, our original experiments were conducted at ruthenium concentrations of 20 mM, to achieve acceptable signal-to-noise levels in the NMR spectra of the catalysts and their propene decomposition products. To probe whether β-H elimination may be masked by rapid bimolecular decomposition under these conditions, we repeated these experiments with nG-C1^Ph^ and nG at 1 mM Ru, using a higher NMR field strength to improve resolution and sensitivity. The proportion of β-H elimination was essentially unaffected for nG-C1^Ph^ (3% over 72 h; Fig. 1b). For nG, it increased sharply, to 95%. We infer that the CAAC catalyst is indeed largely immune to this unimolecular decomposition pathway, whereas for nG, decomposition is dominated by β-H elimination at catalyst concentrations of 1 mM or below.

Unexpectedly, sustained liberation of propenes was observed in the nG experiment over 72 h, although no signals for the precatalyst could be observed after 24 h. We attribute the discrepancy to the continued presence of the metallacyclobutane complex, which goes undetected at RT owing to the breadth of its NMR signals (an indicator of fluxionality or exchange). In assessing complete catalyst decomposition, the intensity of the NMR signals for the organic products is evidently of greater quantitative value than those for the Ru species.

### Density functional theory (DFT) studies of ethylene-triggered decomposition

**Scheme 2:**
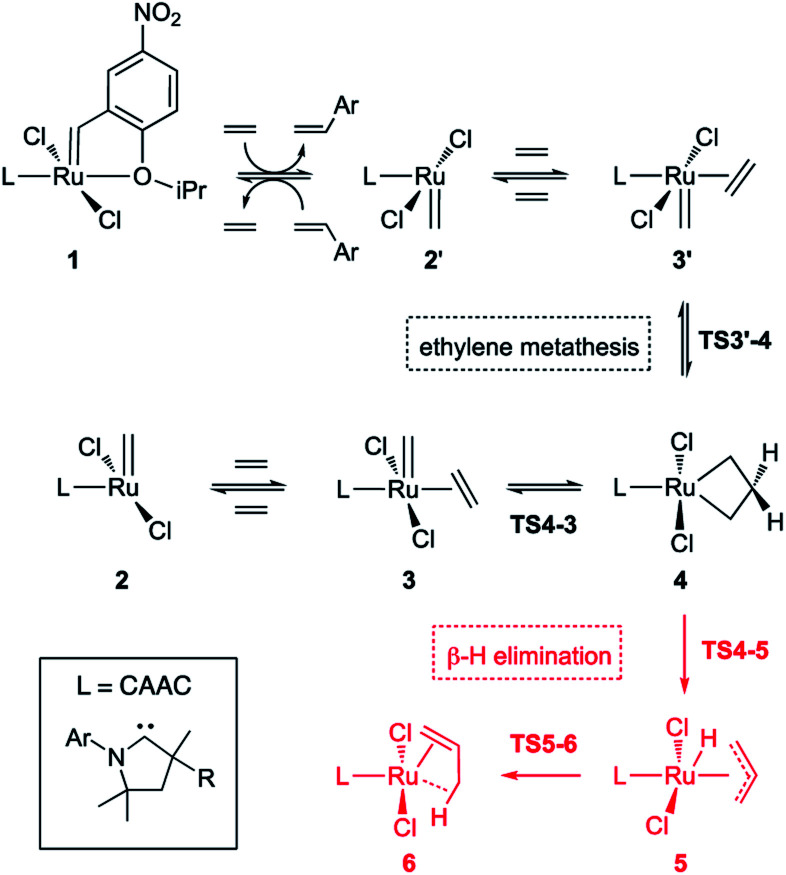
Ethylene self-metathesis and β-H elimination of MCB **4** (L = H_2_IMes, C1^Ph^, C2^Me^). The prime symbol refers to rotamers of the CAAC complexes in which the quaternary CMe_2_ or CMePh site α to the carbene carbon is *syn* to the methylidene. Discussion of the stability and reactivity of the Ru–CAAC rotamers is deferred to a later stage.

In studies of H_2_IMes complexes, the unsubstituted MCB has been identified as much more susceptible to β-H elimination than its substituted analogues.^66^ In the present calculations, we therefore focused on the unsubstituted MCB. Scheme 2 depicts the β-H elimination and ethylene self-metathesis pathways examined. In these metathesis reactions, the position of the alkylidene ‘flips’ in every cycle. Methylidene complex 2′, for example, could be viewed as a rotamer of 2, generated by rotation of the ligand about the Ru–L bond. Carbene rotation, however, has an energetic price. The barriers *via*TS2′-2 are 22.8 or 30.6 kcal mol^−1^ for C1^Ph^ and C2^Me^, respectively (Table S2†), 3.3 or 11.3 kcal higher than the corresponding barriers to ethylene metathesis. Even less likely is carbene rotation in the ethylene complexes, which, with a barrier of 42.7 kcal mol^−1^*via*TS3′-3 for C2^Me^, appears prohibitive. Metathesis is thus the preferred mode of exchange between 2/3 and 2′/3′ species.

This has important implications for the CAAC complexes, owing to their lack of symmetry. In all three square-pyramidal precatalysts 1, the most stable geometry is that in which the alkylidene and the CAAC NAr group are *syn*-disposed (see subsection “The CAAC–Ru rotamers” below). Reaction with ethylene generates the active 14-electron complex 2′, in which the methylidene is anti to the CAAC NAr moiety. Reaction of 2′ with ethylene affords the square-pyramidal π-complex 3′, in which the bound ethylene and the methylidene ligand are mutually perpendicular. Cycloaddition generates the trigonal bipyramidal (TBP) MCB intermediate 4, which upon cycloreversion gives π-complex 3. The latter releases ethylene to form the most stable 14-electron methylidene complex 2, thus completing the unproductive ethylene metathesis reaction. The reverse pathway starts from 2 and ends with 2′.

**Fig. 2:**
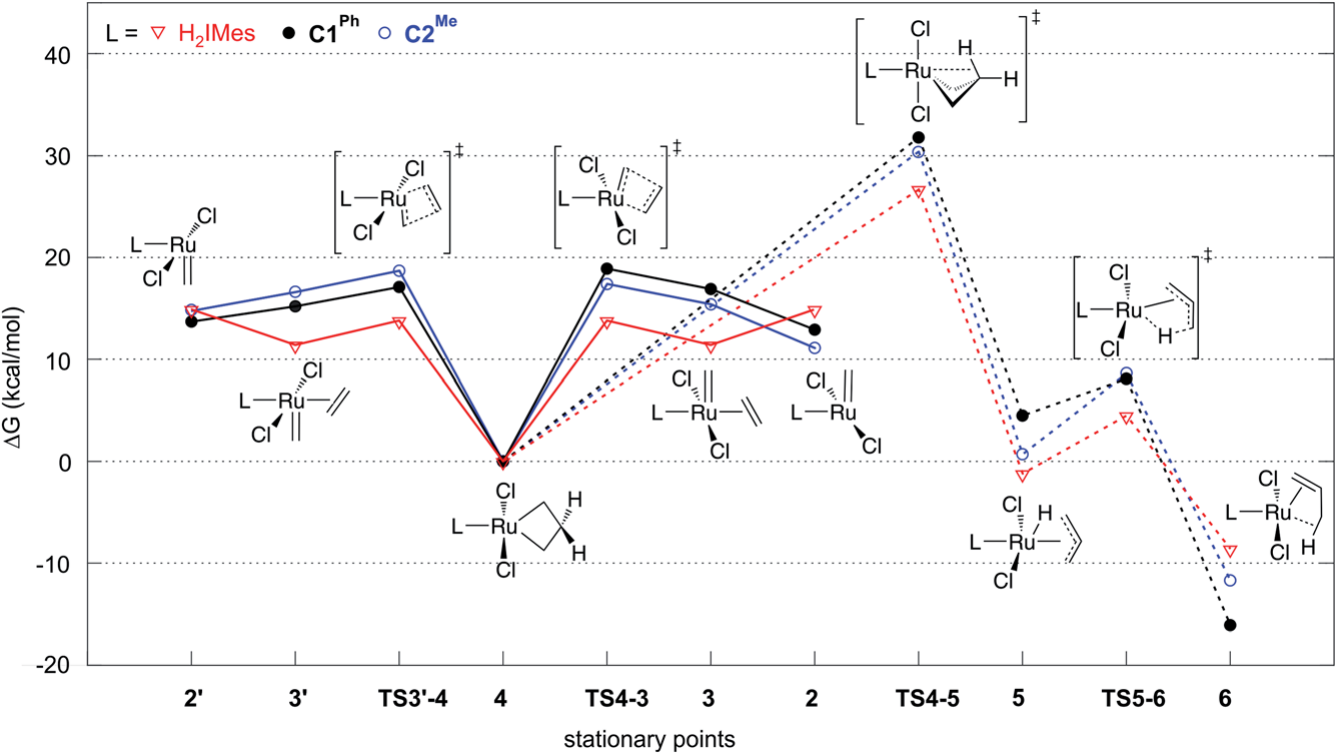
Gibbs free energies of intermediates and transition states in ethylene self-metathesis (solid lines) and β-H elimination (dashed lines). Energies are relative to metallacyclobutane 4, the resting state in ethylene self-metathesis. The individual elementary reactions are given in Scheme 2.

In the absence of competing reactions, this process is repeated until MCB 4 decomposes *via* β-H elimination (Scheme 2, pathway in red; Fig. 2).^83^ The latter reaction involves Ru insertion into the β-C–H bond of the MCB to form allyl–hydride complex 5, followed by hydride transfer to the terminal carbon of the allyl ligand to give π-complex 6, which can then dissociate propene. When a non-isomerizable olefin is used (*e.g.*, ethylene or styrene), propenes are a clear and unambiguous marker for decomposition *via* β-H elimination.^42,43,78^

Reaction of the catalyst precursors with ethylene to form the unsubstituted MCB 4 is exergonic in all cases (by 3.3–5.7 kcal mol^−1^; see Table S2†). Intermediate 4 is the on-cycle resting state. It is also the starting point for catalyst decomposition *via* β-H elimination, and hence the reference point against which free energies are calculated (Fig. 2, Table S2†). β-H elimination to form allyl hydride 5 from 4*via*TS4-5 (dashed lines, Fig. 2) involves a higher activation barrier for CAAC catalysts nG-C1^Ph^ or nG-C2^Me^ than H_2_IMes catalyst nG (by 5.1 or 3.7 kcal mol^−1^, respectively). These DFT-calculated differences in free-energy barriers are sufficiently large that less β-H elimination is expected for CAAC catalysts than for nG, consistent with the much higher proportion of propene decomposition products for the latter.^78^

It may be noted that β-H elimination for any of these MCBs is expected to be slower than for any corresponding acyclic structures, because the requirement for *syn*-coplanarity^47^ noted in the Introduction cannot be met. β-H elimination necessitates a compromise between the required *syn*-coplanar Ru-C_α_-C_β_-H_β_ structure, and the energetically preferred planarity of the MCB ring in 4. Puckering of the MCB ring (the Ru-C_α_-C_β_-C_α_ dihedral angle is in the range 53–56° in TS4-5) enables a reduction in the Ru-C_α_-C_β_-H_β_ dihedral angle by more than 70° on going from 4 (where it is *ca.* 119°) to TS4-5.

Importantly, the calculations also predict that the H_2_IMes catalyst will react with ethylene more readily than do its CAAC counterparts. Whereas ethylene binding to methylidene complexes 2/2′ to form π-complexes 3/3′ is endergonic for the CAAC catalysts, ethylene binding stabilizes the H_2_IMes catalyst; it also lowers the barrier to MCB formation *via*TS4-3 or TS3′-4. We will return to the origin of this difference below. Although somewhat slower MCB formation is predicted for the CAAC catalysts, the barriers to formation of 4 from 2/2′ are negligible. More significantly, the preferred Ru methylidene rotamer 2 is 2.0–3.8 kcal mol^−1^ more stable (*vs.* the resting state 4) for the CAAC complexes than their H_2_IMes analogue. Higher concentrations of the 14-electron methylidene 2 will therefore be present during catalysis for the CAAC catalysts, accounting for their faster decomposition *via* bimolecular coupling.^42,43^

### Decomposition during 1-alkene metathesis

**Scheme 3:**
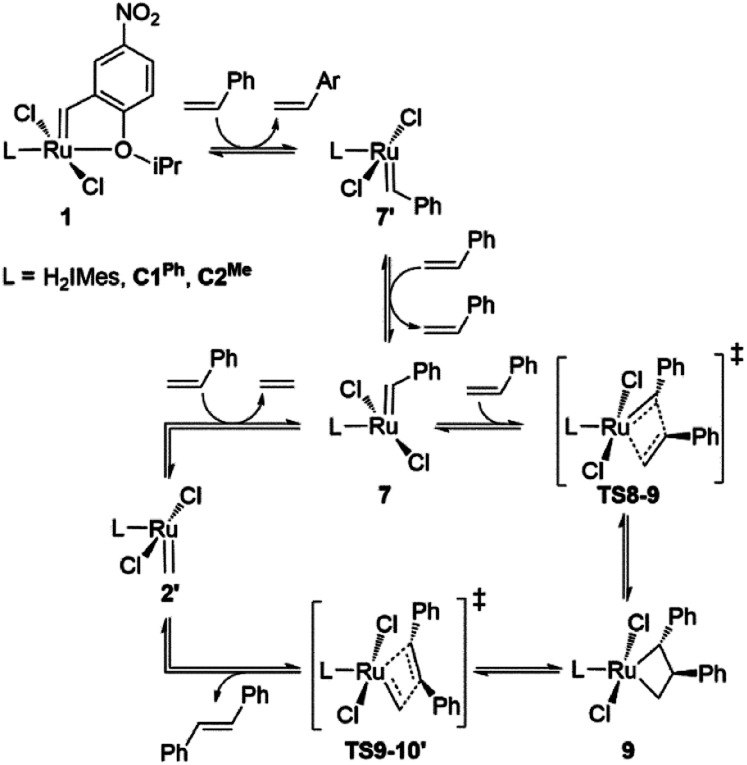
Key intermediates and transition states for styrene self-metathesis. For clarity, only the energetically most favored catalytic cycles, commencing with the 14-electron benzylidene species 7 and ending with 14-electron methylidene species 2′, are depicted.^84^

**Fig. 3:**
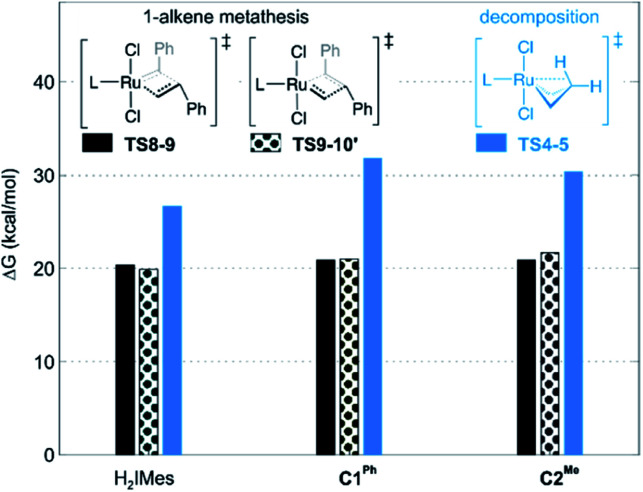
Barriers to 1-alkene metathesis (*via*TS8-9 and TS9-10′) or β-H elimination (*via*TS4-5). Free energies are given relative to the unsubstituted metallacyclobutane 4.

Slower β-H elimination is one clear contributor to the heightened metathesis productivity of the CAAC metallacyclobutane 4, relative to the H_2_IMes derivative. Here we evaluate the relative metathesis productivity of these catalysts, by comparing their barriers to β-H elimination (*via*TS4-5) *vs.* those to self-metathesis of styrene to form trans-stilbene (Scheme 3, Fig. 3). The barriers to metathesis are determined by the transition state for cycloaddition (TS8-9) or retro-addition (TS9-10′). The symmetry of the H_2_IMes ligand results in a single reaction pathway each for metathesis and β-H elimination. For the unsymmetrical CAAC complexes, four competing pathways are operative for each of these reactions (see the ESI† for details). The two energetically favored pathways (corresponding to Pathways 3 and 4 in Table S2†) involve cycloaddition transition states TS8-9, with the benzylidene-derived phenyl moiety *syn* to the NAr group. These rotamers imply a catalytic cycle commencing with 14-electron benzylidene species 7 and ending with 14-electron methylidene species 2′, as depicted in Scheme 3. In the preferred, lowest-barrier pathway, the benzylidene moiety is oriented away from the quaternary phenyl substituent of C1^Ph^, or the isopropyl substituent of C2^Me^. The corresponding transition state for retro-addition is TS9-10′, in which the methylidene moiety is *syn* to the quaternary site.

A smaller energy difference between the barriers to β-H elimination (TS4-5*vs.*4) and styrene metathesis (TS8-9 or TS9-10′*vs.*4, whichever is the less stable) is seen for the H_2_IMes catalyst than the CAAC catalysts. Specifically, the difference is 6.3 kcal mol^−1^ for nG, *vs.* 10.4 or 8.7 kcal mol^−1^, respectively, for nG-C1^Ph^ or nG-C2^Me^: see Fig. 3. The H_2_IMes catalyst thus has a lower energetic ‘buffer’ against β-H elimination from the unsubstituted MCB, consistent with its greater susceptibility to this decomposition pathway.^78^

All catalysts studied exhibited a higher barrier to styrene self-metathesis than ethylene metathesis, presumably resulting from both the steric bulk^85^ and the electron-withdrawing properties of the phenyl substituent.^86^ For the H_2_IMes catalyst nG, the difference is 6.6 kcal mol^−1^, *vs.* 2.6 or 3.1 kcal mol^−1^ for nG-C1^Ph^ or nG-C2^Me^, respectively. This reinforces the more facile reaction of nG with ethylene discussed above. The CAAC catalysts are thus predicted to have a greater bias toward 1-alkene metathesis. Their lower reactivity toward ethylene is expected to increase productivity at high 1-alkene conversions, by limiting unproductive cycling with the ethylene co-product of metathesis, which opens the door to decomposition of the unsubstituted MCB. Conversely, at low conversions, 1-alkene coupling should be faster with the H_2_IMes catalyst, which has the lowest calculated barrier to styrene self-metathesis. This is consistent with the observed bias of NHC catalysts toward self-metathesis at low conversions in ethenolysis experiments.^74^

### Factors determining the rates of metathesis and β-H elimination

**Fig. 4:**
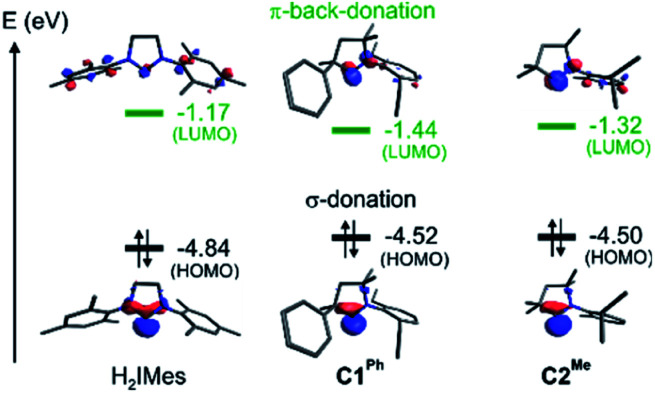
Energies and shapes, given as isosurface plots of ± φ(*x*,*y*,*z*) = 0.08 a.u., of the DFT (Kohn–Sham) frontier orbitals most relevant to the carbene σ-donor and π-acceptor properties.

To probe the stereoelectronic factors responsible for the outstanding productivity and robustness of the CAAC catalysts, we examined properties of the carbene ligands and their Ru complexes. In general, CAAC ligands are known to have less stable σ-donor orbitals and more stable π-acceptor orbitals than corresponding cyclic diaminocarbenes, and therefore to be both better σ-donors and π-acceptors.^87–92^ To obtain a first, qualitative comparison of the donor/acceptor properties specific to the three leading carbenes under study, we calculated the energies of their frontier orbitals, focusing on those with the shape and symmetry appropriate for bonding interactions with the metal (Fig. 4).^93,94^ Carbene σ-donation is dominated by the highest-energy occupied molecular orbital (HOMO), which is centered on the carbene carbon atom and has σ-symmetry with respect to the metal–carbene bond. The lowest unoccupied molecular orbital (LUMO) is the corresponding unoccupied carbene frontier orbital with π-symmetry. The energies of the frontier orbitals suggest that the two CAAC ligands should have similar donor/acceptor properties, but that both should be better σ-donors and better π-acceptors than H_2_IMes.

Critical in metathesis is the impact of these differences on the frontier orbital energies in the unsubstituted MCB. Following the procedure described in ref. 95, natural bond orbital (NBO) analysis^96^ of 4 confirmed stronger σ-donation as well as π-back-donation for the CAAC complexes (donation/back-donation = 0.66/0.20 and 0.67/0.21 electrons for C1^Ph^ and C2^Me^, respectively) than for the H_2_IMes complex (0.57/0.15 electrons). The stronger orbital interactions for the CAAC ligands give rise to stronger bonds to ruthenium: the calculated bond dissociation free energies are *ca.* 5 kcal mol^−1^ higher (42.8 and 43.3 kcal mol^−1^ for C1^Ph^ and C2^Me^, respectively) than for H_2_IMes (38.0 kcal mol^−1^).

**Fig. 5:**
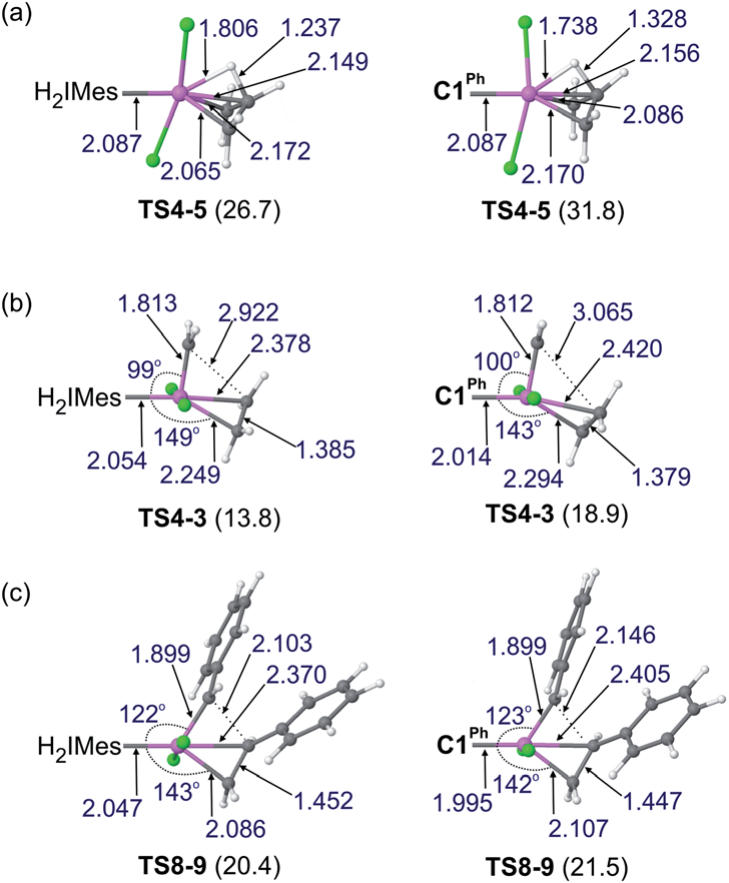
Optimized geometries for transition-state species corresponding to key barriers for: (a) β-H elimination; (b) ethylene self-metathesis; (c) styrene self-metathesis. Ru: pink; Cl: green; C: grey; H: white. Shown are selected bond distances (Å), bond angles (°), and (in parentheses) the Gibbs free energy (kcal mol; in CHCl_3_) relative to 4, the most stable reaction intermediate.

**Fig. 6:**
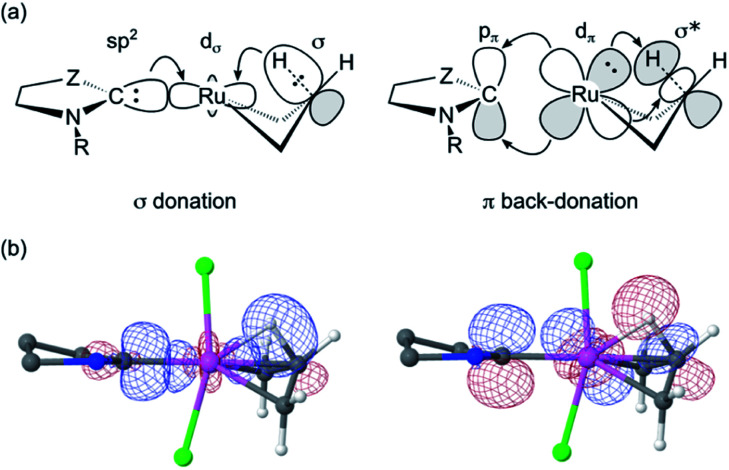
(a) Idealized representation of competing molecular orbital interactions affecting the energy of TS4-5 (see Table 1 for donor–acceptor interaction energies). For clarity, only the most relevant atoms are shown. Z = NAr or CRR’. (b) Combined isosurface representations (cutoff = 0.09 a.u.) of the three natural bond orbitals involved in the competition for σ-donation (left) and π-back-donation (right) in TS4-5 for C1^Ph^. Ru: pink; Cl: green; C: grey; N: blue; H: white. Hydrogen atoms and substituents of C1^Ph^ have been omitted for clarity.

Importantly, the stronger metal–carbene orbital interactions result in a higher trans influence and trans effect for the CAAC ligands.^60^ In addition to weakening trans-positioned bonds in equilibrium geometries, the strong orbital interactions of the CAAC ligands have critical kinetic consequences. In transition state TS4-5, the rupturing C_β_–H bond approaches the ruthenium center trans to the carbene (Fig. 5a and 6a). It thus necessitates mutual trans interactions of two high trans-effect ligands, the carbene and the nascent hydride. This is more costly for a CAAC than a H_2_IMes MCB. The higher barrier to β-H elimination for the former is thus proposed to arise from the higher trans effect characteristic of the CAAC carbenes, relative to H_2_IMes.

The proposed role of the trans effect is confirmed by an NBO-based second-order perturbation analysis of donor–acceptor interactions in TS4-5 (Fig. 6; Table 1). The carbene lone pair and the β-C–H bond compete for σ-donation to the same Ru acceptor orbital. Simultaneously, the carbene π-acceptor orbital and the antibonding β-C–H and Ru–C orbitals compete for the same Ru lone pair. That is, the increased competition for donation and back-donation at the transition state retards β-H elimination for the CAAC complexes.

**Table 1:** Donor–acceptor interaction energies of NBO-based second-order perturbation analysis of TS4-5

Carbene	Donor–acceptor interaction energy^a^ (kcal mol^−1^)			
	sp^2^ → d_σ_	σ → d_σ_	d_π_ → p_π_	d_π_ → σ*
H_2_IMes	141.3	50.4	37.8	6.9
C1^Ph^	143.2	61.9	19.6	7.6
C2^Me^	135.2	63.7	17.3	10.0

a The orbital labels are defined in Fig. 6.

Computational studies of alkyl cross-coupling *via* group 10 catalysts describe similar retarding effects where β-H elimination occurs trans to dative ligands of strong trans effect.^52,53^ Likewise, high trans-effect anionic ligands (X) have been found to retard oxidative addition of methane and ammonia at the site trans to X in Ir(i) complexes.^97^ A related, more indirect, effect on the rate of β-H elimination has been observed in olefin metathesis for d^0^ molybdenum and tungsten catalysts, in which higher barriers to β-H elimination have been calculated for oxo-stabilized MCBs than for their imido analogues.^58^ The d^0^ metal catalysts preferentially undergo β-H elimination after isomerizing to a square-pyramidal MCB, in which the C_β_–H bond approaches the metal cis, rather than trans, to the imido or oxo ligand. The latter ligands are trans to one of the rupturing M–C bonds of the MCB, and hence destabilize the β-H elimination transition state^58^ (particularly the oxo ligand, which has a stronger trans effect).^60^ A similar role of the oxo ligand in retarding β-H elimination was recently described for vanadium–oxo olefin metathesis catalysts.^57^ Taken together, all these examples suggest that high trans-effect ligands offer an important general strategy, valid across multiple catalytic manifolds, to suppress β-H elimination.

In ruthenium-catalyzed olefin metathesis, the capacity of the high trans-effect carbene ligand to inhibit β-H elimination can now be explicitly identified as a key contributor to the remarkable productivity of the CAAC catalysts. The high trans effect also has negative consequences, however, as we recently demonstrated: the labilizing effect of the CAAC ligand increases the concentration of the four-coordinate methylidene species 2/2′, and thus promotes decomposition *via* bimolecular coupling.^42^ These two decomposition pathways, long viewed as independent, can now be recognized as opposing responses to a single underlying phenomenon, which originates in strong ligand binding. This is particularly important given the transformative role played by strong carbene donicity in Ru-catalyzed olefin metathesis.^30,98^

In addition to retarding β-H elimination, the high trans influence of the CAAC ligands destabilizes the ethylene π-complexes 3/3′, in which ethylene (also a high trans-influence ligand) binds trans to the carbene. “Early” transition states are seen en route to the MCB 4 (TS3′-4 and TS4-3). That is, the TS geometries closely resemble those of the square-pyramidal π-complexes (Fig. 5b): the ethylene ligand is still far from parallel to the Ru=CH_2_ bond,^99–101^ and the double bond is nearly intact, with very little interaction with the methylidene, as suggested by a Wiberg bond index (WBI, a bond-order measure)^102^ of >1.5 for the ethylene C_α_–C_β_ bond, and <0.15 for its C_β_–C_α_ bond to the methylidene (see Fig. S8†). In consequence, the trans influence that affects the ethylene π-complexes is also manifested in a trans effect. The latter kinetic effect retards ethylene self-metathesis by destabilizing the early, π-complex-like transition states (behavior analogous to that seen in β-H elimination *via*TS4-5 above). The barrier to metathesis of ethylene hence increases by *ca.* 5 kcal mol^−1^ for the CAAC complexes, compared to the H_2_IMes analogue.

Styrene self-metathesis, in contrast, is found to proceed *via* a “late” transition state (*e.g.*, TS8-9, Fig. 5c). The geometry of the latter resembles that of the MCB intermediate 4: it shows a significantly elongated styrene double bond (1.45 Å, compared to 1.35 Å in free styrene), and clear interaction with the benzylidene carbon, with a C–C distance only slightly longer than 2 Å. Ruthenacyclobutane intermediates, and transition states (such as TS8-9) that resemble such intermediates, have distorted TBP geometries. The MCB ring lies in the trigonal plane, and no ligand is bound trans to the carbene, unlike the π-complexes 3/3′ and the early transition states TS3′-4 and TS4-3. Within the trigonal plane, strong bonds exist between the Ru center, the carbene ligand, and the MCB C_α_ atoms. Positioned trans to the carbene, but with a Ru–C_β_ distance 0.28–0.51 Å longer than the Ru–C_α_ bonds, is the β-carbon atom. The latter interacts weakly with Ru, unsurprisingly given that it already engages in four σ-bonds (MCB intermediates) or is well on the way to forming the fourth σ-bond (TS8-9 and TS9-10′). The Ru–C_β_ Wiberg bond indices of these late transition states (0.11–0.14; see Fig. S8†) are a small fraction of those calculated for the strong metal–ligand bonds in the trigonal plane, and only *ca.* half those of Ru–C_β_ bonds of the early transition states TS3′-4 and TS4-3.

**Fig. 7:**
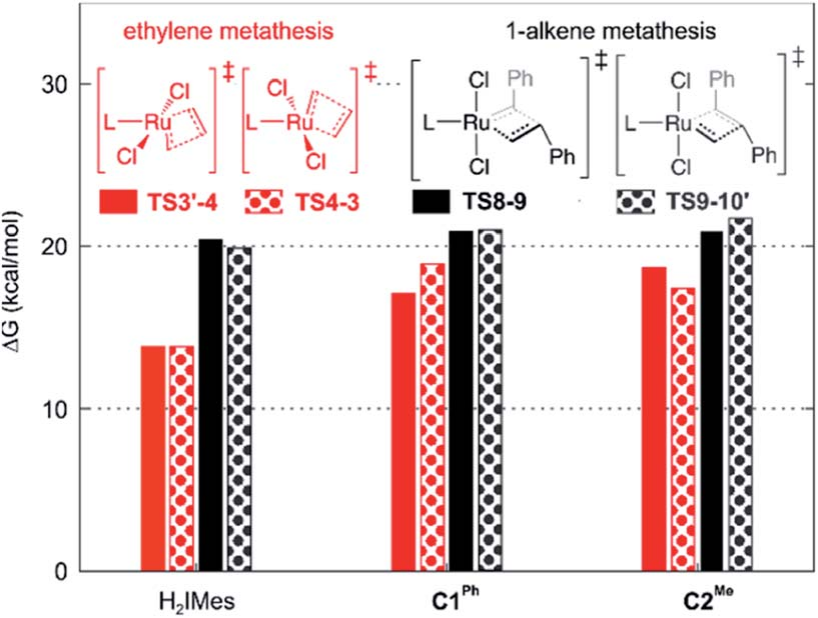
Calculated barriers to metathesis of ethylene *vs.* styrene (in kcal mol^−1^, *vs.*4).

Styrene metathesis is thus little affected by the carbene trans effect, in contrast to β-H elimination and ethylene self-metathesis. Indeed, the barriers to styrene metathesis are within 1.3 kcal mol^−1^ for all three catalysts. All are higher than the barriers to ethylene self-metathesis, as noted above, but the energetic preference for ethylene self-metathesis is higher for the H_2_IMes catalyst than its CAAC analogues (by 6.6, 2.1 or 3.0 kcal mol^−1^ for nG, nG-C1^Ph^ and nG-C2^Me^, respectively); see Fig. 7.

These differences in barrier heights imply that the CAAC catalysts are less susceptible to non-productive cycling with ethylene. The superior productivity of the CAAC catalysts thus arises not merely from their resistance to β-H elimination, but from their improved selectivity for 1-alkenes, relative to ethylene. The slightly reduced preference for ethylene self-metathesis calculated for nG-C1^Ph^, *vs.*nG-C2^Me^ (a difference of 0.9 kcal mol^−1^) is presumed to reinforce the positive effects of the quaternary phenyl substituent on metathesis productivity.^79,103^

Again, the favorable properties of the CAAC ligands originate in the high trans effect exerted by these carbenes in the transition states for β-H elimination and ethylene self-metathesis.

In contrast, and perhaps surprisingly, steric differences between the three carbenes do not seem to play a significant role. Their overall steric features are similar, as judged from both the buried volumes^104^ calculated for 4 (%*V*_bur_ = 33.6%, 36.2%, and 34.3% for H_2_IMes, C1^Ph^, and C2^Me^, respectively) and the natural steric exchange repulsion energy^105^ calculated between the carbene, the methylidene, and the chloride ligands in the methylidene complexes 2/2′ (28.8/28.8 kcal mol^−1^, 33.0/35.1 kcal mol^−1^, and 30.9/28.8 kcal mol^−1^, for H_2_IMes, C1^Ph^, and C2^Me^, respectively; see the ESI for details). From both methods, C1^Ph^ appears only slightly bulkier than H_2_IMes and C2^Me^. In short, neither the differences in overall bulk, nor those in spatial steric distribution (as represented by steric maps;^104^ see the ESI†), appear sufficient to account for the robustness and productivity of the two CAAC catalysts.

### The CAAC–Ru rotamers

**Fig. 8:**
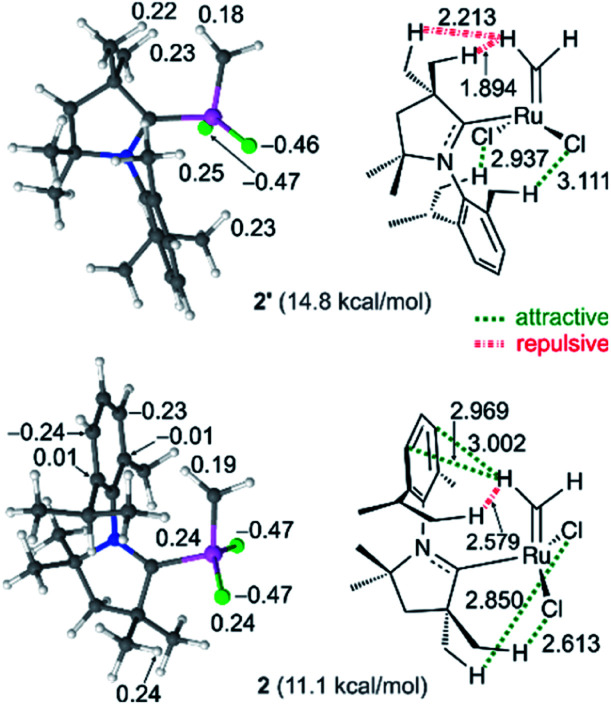
Natural charges (*e*) of selected atoms appear in the ball-and-stick models of the optimized geometries (left). Repulsive and attractive interactions, and selected atomic distances (Å), of rotamers 2/2′ of nG-C2^Me^ (right). Ru: pink; Cl: green; C: grey; N: blue; H: white. Given in parentheses are the free energies *vs.*4.

As a final point, the relative stability and reactivities of the various rotamers – an inevitable complication arising from the asymmetry of the CAAC ligands – deserves some comment. Within the C2^Me^ catalyst system, rotamer 2 (in which the methylidene ligand is anti to the quaternary site flanking the carbene carbon) is more stable than rotamer 2′ by 3.7 kcal mol (see Fig. 2), despite the lower steric exchange repulsion (by 2.1 kcal mol^−1^) calculated for the latter. The explanation lies in overriding electronic effects (Fig. 8). Whereas 2′ is destabilized by strong electrostatic repulsion between the methylidene and the nearby quaternary methyl groups, 2 is stabilized by attraction between the quaternary methyl groups and the chloride ligands. Similar, but weaker, attractive interactions between the chloride ligands and the NAr alkyl groups stabilize 2′. In 2, the electrostatic repulsion between the NAr alkyl groups and the methylidene is much weaker than the corresponding repulsion from the quaternary methyl groups in 2′ and is also offset by stabilizing polar CH–π interactions^42,106^ between the methylidene and the aromatic NAr group. The calculations thus predict that in the most stable geometry 2, the NAr group is *syn* to the alkylidene. In fact, within the C2^Me^ catalyst system, this rotamer was found to be preferred for all the alkylidene species and ethylene self-metathesis transition states studied. π-Face donation from the C_ipso_ atom of the NAr to the alkylidene carbon atom^107–109^ is also expected to stabilize the *syn* conformation of alkylidenes parallel to the NAr plane, such as in the ethylene complex 3, and in corresponding transition state TS4-3.

In contrast, the aromatic quaternary substituent of nG-C1^Ph^ engages in attractive polar CH–π interactions^42,106^ with the alkylidene, stabilizing those rotamers in which the quaternary CAAC site is *syn* to the alkylidene. The latter represent the most stable ethylene complex (3′) and corresponding transition state (TS3′-4) leading to 4. Consistent with these contrasting predicted rotamer stabilities, NOESY-NMR analysis revealed interactions between the [Ru]=C*H*Ar proton and the quaternary phenyl group for the Piers-class catalyst P-C1^Ph^ (Chart 1; Fig. S4†), but not for P-C2^Me^. Likewise, [Ru]=C*H*_2_–phenyl interactions could be detected for a pyridine-stabilized derivative of 2′ for C1^Ph^, although rapid decomposition precluded unambiguous interpretation of the spectrum for the C2^Me^ analogue.

This phenyl-induced stabilization of a rotamer that is destabilized in nG-C2^Me^ facilitates metathesis by nG-C1^Ph^. Facile alkylidene ‘flipping’ promotes engagement of both rotamers in catalysis, and their relative stability has a positive impact on the transition states connecting them to the rest of the catalytic cycle. For example, the barrier to styrene self-metathesis for nG-C1^Ph^ is lower by 0.7 kcal mol^−1^ compared to nG-C2^Me^, thereby further improving the selectivity for 1-alkene *vs.* ethylene metathesis.

## Conclusions

Unlike their NHC predecessors (notably those bearing an H_2_IMes ligand), the ruthenium–CAAC metathesis catalysts studied are essentially immune to decomposition *via* β-H elimination from the metallacyclobutane. Calculations predict a higher barrier to β-H elimination in the CAAC systems, consistent with the distinctions in behavior observed experimentally. Also predicted is higher selectivity for metathesis of styrene, *vs.* unproductive ethylene self-metathesis, relative to the Ru–NHC catalysts. The poorer selectivity of the latter (that is, their tendency to engage in metathesis of ethylene), in conjunction with the greater vulnerability to β-H elimination of the unsubstituted MCB thus formed, represent a lethal combination of effects that explains the lower metathesis productivity of the popular NHC catalysts relative to the emerging CAAC systems.

The higher barriers to β-H elimination and ethylene self-metathesis calculated for the CAAC catalysts originate in the stronger carbene–metal orbital interactions. These interactions destabilize both intermediates (trans influence) and transition states (trans effect) involving competing orbital interactions trans to the carbene, notably TS4-5 (β-H elimination) and TS3′-4/TS4-3 (ethylene self-metathesis). Thus, both the greater resistance to β-H elimination and the improved selectivity for productive 1-alkene metathesis of the CAAC catalysts are due to the higher trans effect of this carbene class.

The findings above add ruthenium olefin metathesis catalysts to the systems for which high trans-influence ligands have been found to retard β-H elimination,^52,53,57,58^ underlining the generality and the scope of this ligand effect in catalysis. Crucially, for ruthenium, the impact is manifested in lower rates of reaction (both β-H elimination and ethylene cycloaddition) taking place directly trans to the ligand.

The high trans influence and trans effect are clearly critical to the breakthrough success of the CAAC ligand family. However, these properties also have a profound negative consequence, greatly enhancing the susceptibility to bimolecular coupling of 4-coordinate methylidene species 2/2′.^42^ The positive impact of a strong trans-effect ligand in limiting β-H elimination is thus offset by its potent accelerating effect on bimolecular decomposition. These two decomposition pathways have long been known to limit productivity in olefin metathesis. They are here revealed as distinct, antinomic, responses to a single underlying phenomenon. Reconciling these opposing effects is a clear priority for catalyst design. More robust and productive olefin metathesis catalysts will aid in expanding applications in demanding contexts. One specific, compelling goal is the development of stereoselective catalysts resistant to β-H elimination, including as-yet-undiscovered stereoselective CAAC catalysts.

## Data availability

The complete computational data is available from the ioChem-BD repository,^110^*via*https://doi.org/10.19061/iochem-bd-6-113.

## Author contributions

DEF and VRJ designed and supervised the project. GO performed the DFT calculations, with initial contributions from MF. MF handled, organized, and stored the computational data (see Data availability). DLN performed the experiments. All authors analyzed the computational and experimental results. GO wrote the initial draft. DEF and VRJ wrote the final manuscript, with contributions from GO, MF, and DLN. All authors have approved the final manuscript.

## Conflicts of interest

There are no conflicts to declare.

## Supplementary Material

SC-013-D2SC00855F-s001

SC-013-D2SC00855F-s002
