# Supplementary material for: The Janus face of high trans-effect carbenes in olefin metathesis: gateway to both productivity and decomposition
Source: Chem Sci. 2022 Mar 22;13(18):5107–17. doi: 10.1039/d2sc00855f (PMC9093171; doi:10.1039/d2sc00855f)
Supplement: SC-013-D2SC00855F-s001 [file SC-013-D2SC00855F-s001.pdf]

Electronic Supplementary Material (ESI) for Chemical Science.
This journal is © The Royal Society of Chemistry 2022

Electronic supplementary information for:

The Janus face of high trans-effect carbenes in olefin metathesis: gateway to both productivity and decomposition

Giovanni Occhipinti,[a]* Daniel L. Nascimento,[b] Marco Foscato,[a] Deryn, E. Fogg,[a,b]* and Vidar R. Jensen[a]*

[a]Department of Chemistry, University of Bergen, Allégaten 41, N-5007 Bergen, Norway. [b]Center for Catalysis Research & Innovation, and Department of Chemistry and Biomolecular Sciences, University of Ottawa, Ottawa, Canada K1N 6N5.

Table of Contents

1 Experimental procedures **S2**

1.1 Representative procedure for quantifying β-elimination at 1 mM Ru S2

1.2 Representative procedure for NOESY experiments S2

2 Computational methods **S10**

2.1 Model building S10

2.2 Geometry optimization S10

2.3 Single-point energy calculations S10

2.4 Calculation of Gibbs free energies S11

2.5 Natural bond orbital (NBO) analyses S11

2.6 Calculated steric volumes S12

3 Computational results **S12**

3.1 Molecular geometries S12

3.2 Natural bond orbital (NBO) analyses S12

3.3 Calculated steric volumes S15

3.4 Calculated total and free energies S16

4 References **S21**

1 Experimental procedures

1.1 Representative procedure for quantifying β-elimination at 1 mM Ru

A mixture of green **nG** (11 mg, 0.017 mmol) and DMT (5 mg, 0.026 mmol, 1.5 equiv) was dissolved in 1.00 mL $CDCl_3$ to give a 16.8 M stock solution of precatalyst. An 80 μL aliquot (1.34 μmol **nG**) was transferred to a J-Young NMR tube and diluted with 500 μL $CDCl_3$. The initial ratio of **nG**:IS ratio was measured, after which the NMR sample was returned to the glovebox, where $CDCl_3$ (440 μL) and styrene (307 μL, 2.68 mol, 2 000 equiv) were added to give a solution that was 1 mM in Ru. The NMR tube was attached to a mechanical rotator set to 10 rpm to maintain steady mixing over a 72 hour period. The timer was started immediately, and spectra were collected periodically (see Fig. 1 in main text). For the initial and final spectra, see Fig. S1.

nG-C1^{Ph}: As above, with a stock solution of 16 mg **nG-C1^{Ph}** (0.023 mmol) and 5 mg DMT (0.026 mmol, 1.1 equiv) in 1.00 mL $CDCl_3$ (22.6 mM Ru). For initial and final spectra, see Fig. S2.

1.2 Representative procedure for NOESY experiments

Piers-class catalysts and their pyridine-stabilized methylidene derivatives were synthesized by the reported method.[1] Ca. 10 mg of each was transferred to a J-Young NMR tube and dissolved in 0.5 mL $CDCl_3$. ^{1}H NOESY NMR spectra were collected over 4 h at RT (Piers complexes) or −40 °C (methylidene complexes).

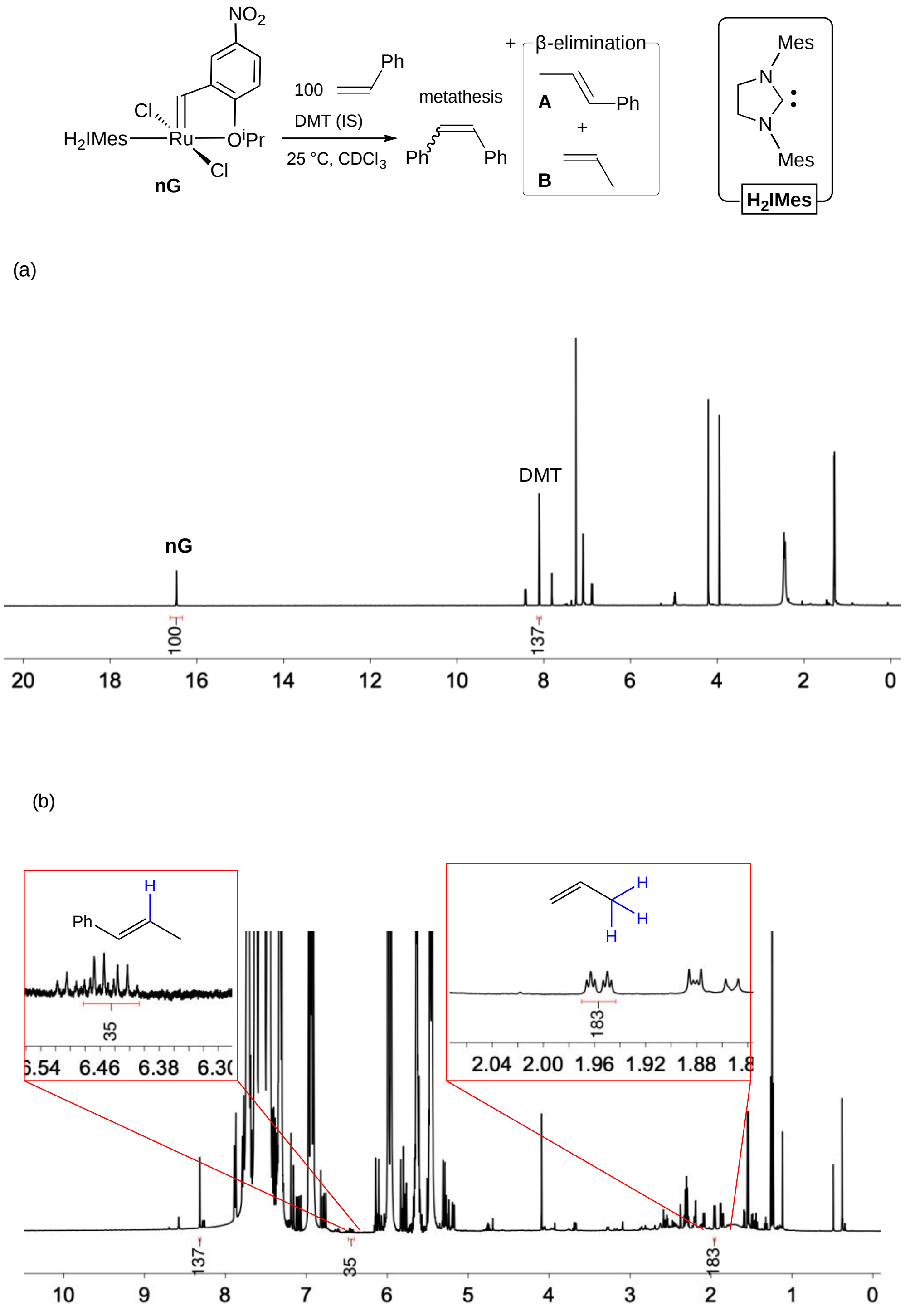

Fig. S1 ^{1}H NMR spectra (500 MHz, $CDCl_3$) confirming that decomposition of **nG** occurs solely by via β-hydride elimination at 1 mM Ru. (a) Initial spectrum (t_0), showing the integration of the alkylidene signal ([Ru]=C*H*Ar) for catalyst **nG** relative to internal standard (dimethyl terephthalate, DMT). (b) Spectrum at 72 h, showing generation of ca. 95% propenes, the marker for β-hydride elimination (35% β-methylstyrene, **A**; 61% propene, **B**). The balance is attributed to partial loss of volatile propene to the headspace over 72 h. For authentic samples of **A** and **B**, see Fig. S3.

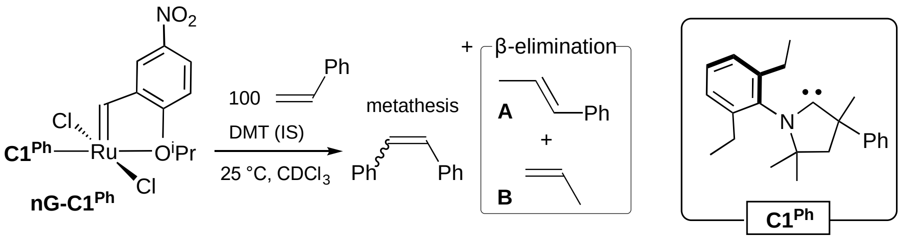

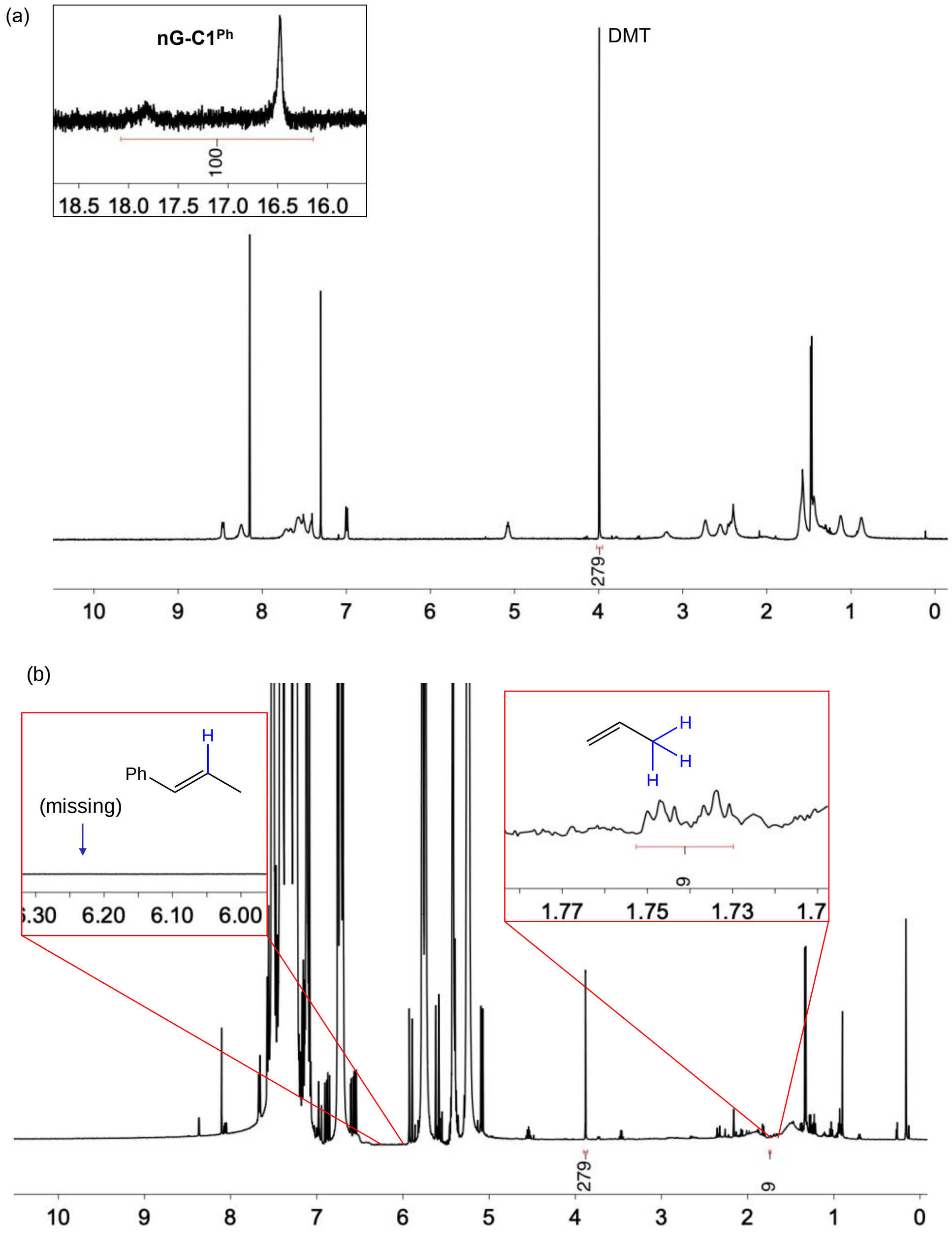

Fig. S2 ^{1}H NMR spectra (500 MHz, $CDCl_3$) showing near-zero decomposition via β-hydride elimination for **nG-C1^{Ph}** at 1 mM Ru. (a) Initial spectrum (t_0); showing the integration of the alkylidene signals ([Ru]=C*H*Ar) for catalyst **nG-C1^{Ph}** relative to DMT as internal standard. (b) Spectrum at 72 h, showing generation of ca. 3% propene **B**: no β-methylstyrene **A** is observed.

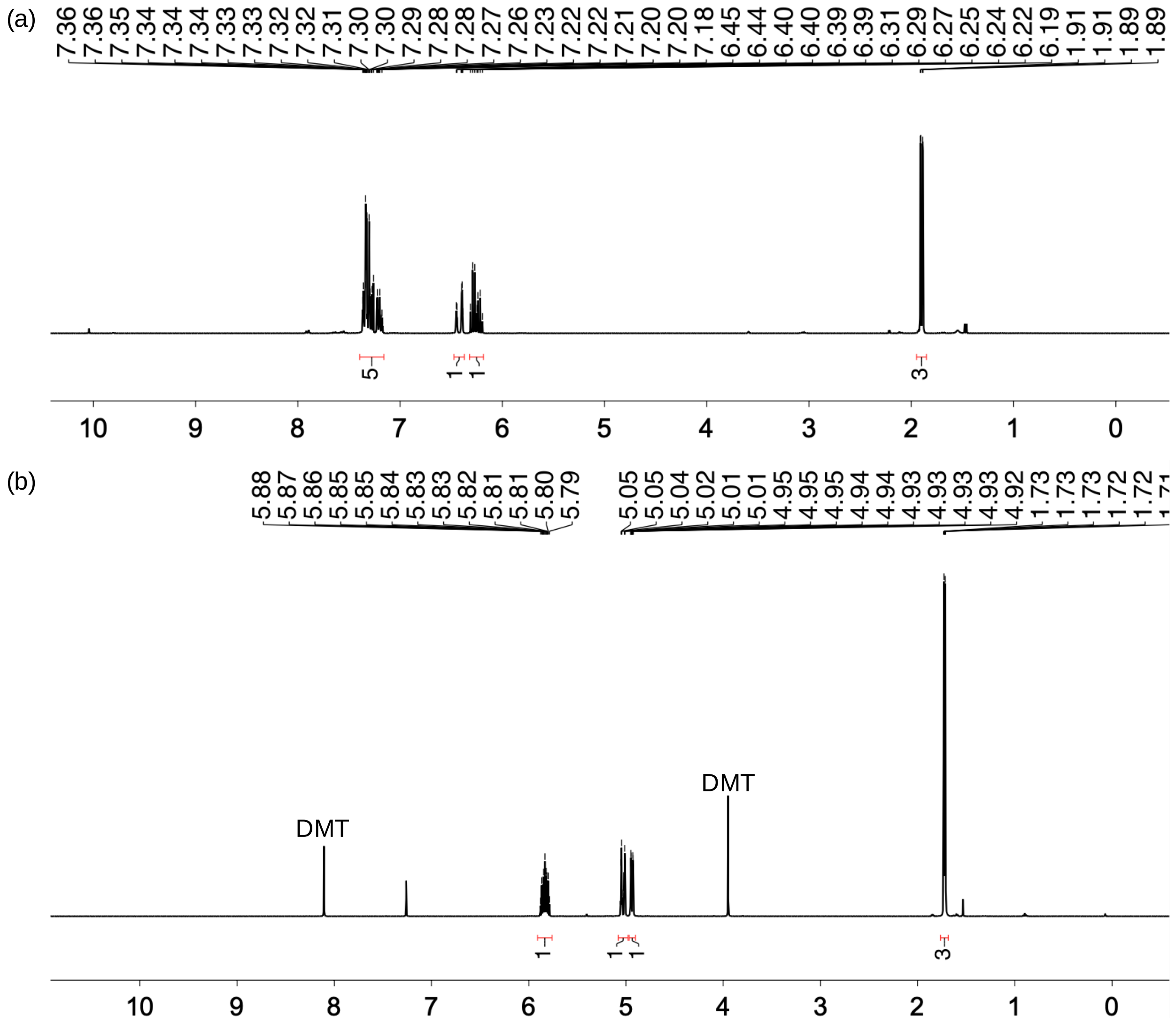

Fig. S3 ^{1}H NMR spectra (500 MHz, $CDCl_3$) of authentic samples of propenes. (a) β-Methylstyrene, **A**. (b) Propene, **B** and internal standard DMT.

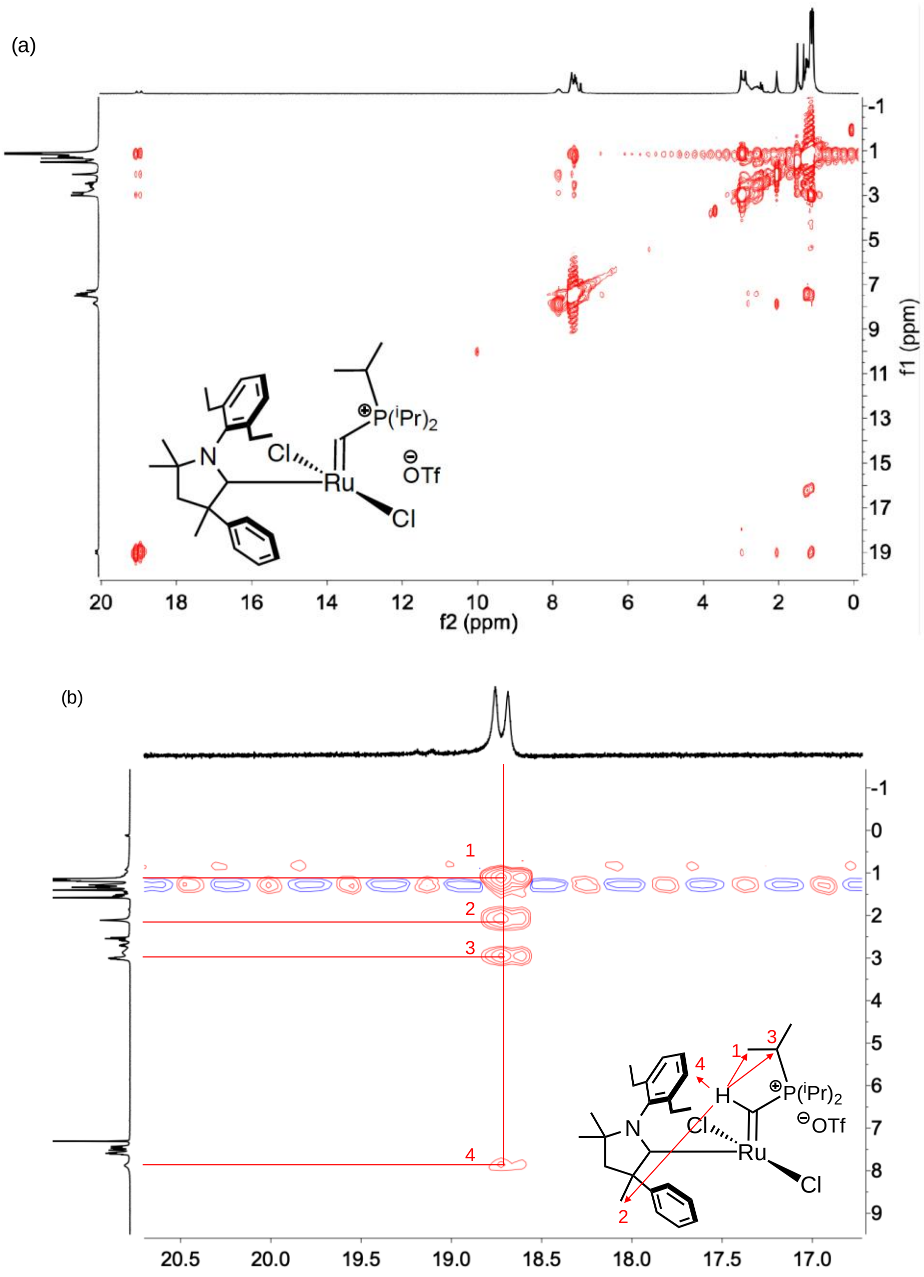

Fig. S4 ^{1}H NOESY-NMR spectra (500 MHz, $CDCl_3$) for Piers catalyst **P-C1^{Ph}**. (a) Full NOESY spectrum. (b) Expansion, showing NOE interactions of the alkylidene proton ([Ru]=C*H*P^{i}Pr) with substituents flanking both sides of the carbene carbon, consistent with the predicted electrostatic interactions for the **C1^{Ph}** ligand. As with the methylidene derivatives (e.g. **2'**, **2**; see main text), two rotamers are visible, arising from rotation of the CAAC ligand about the Ru–L bond. (Also observed are interactions with the isopropyl methine and methyl protons of the adjacent P^{i}Pr group). Interactions of the alkylidene proton and the NAr *o*-Et protons are also presumed, but are masked by signal overlap in this crowded part of the alkyl region (1–1.5 ppm).

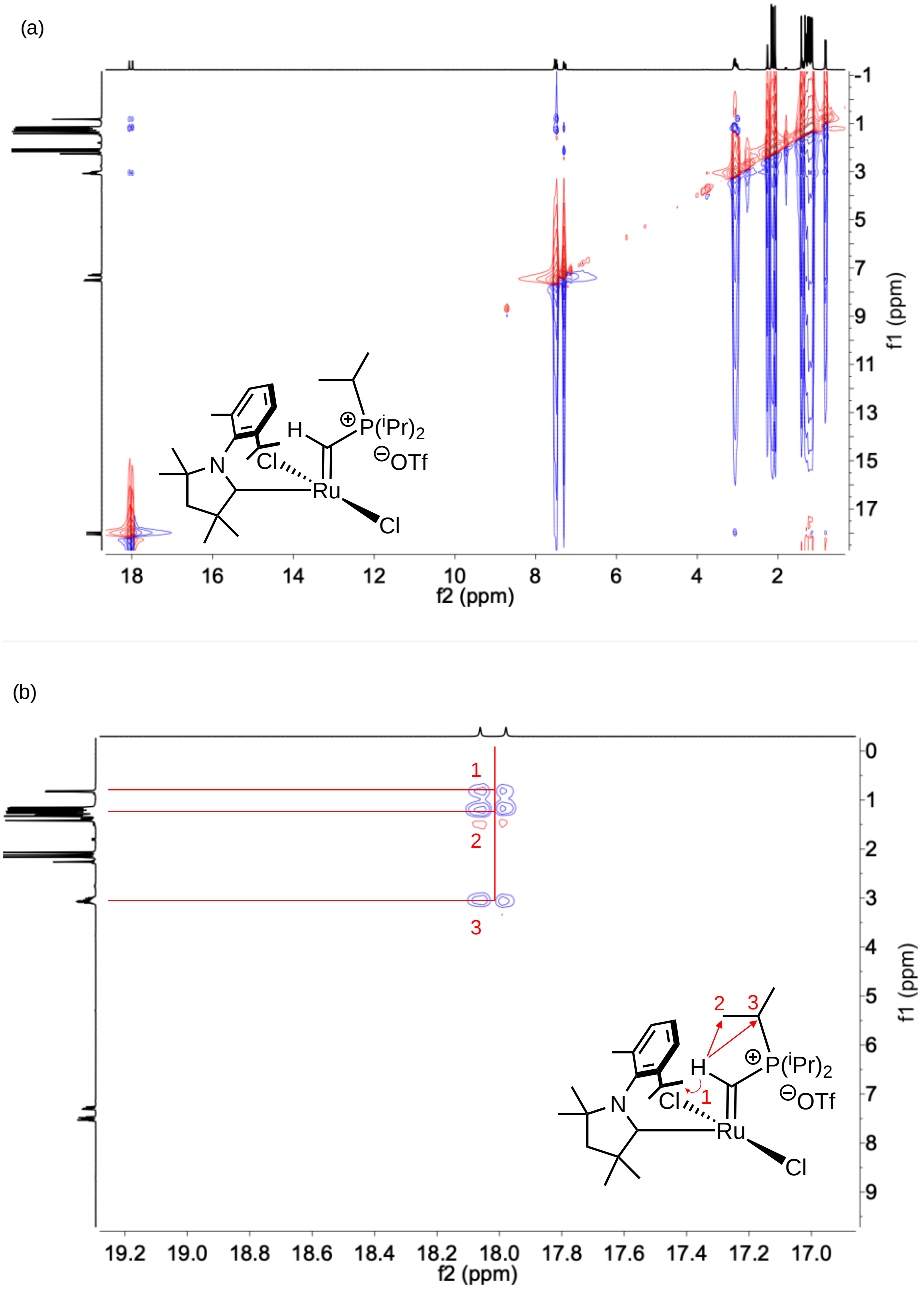

Fig. S5 ^{1}H NOESY-NMR spectra (500 MHz, $CDCl_3$) for Piers catalyst **P-C2^{Me}**. (a) Full NOESY spectrum. (b) Expansion, showing NOE interactions of the alkylidene proton ([Ru]=C*H*P^{i}Pr) only with the NAr group flanking the carbene carbon (specifically, the isopropyl methyl groups). Not seen is an interaction with the CMe_2 protons, consistent with the predicted electrostatic interactions for the **C2^{Me}** ligand. (Also observed are interactions with the isopropyl methine and methyl protons of the adjacent P^{i}Pr group).

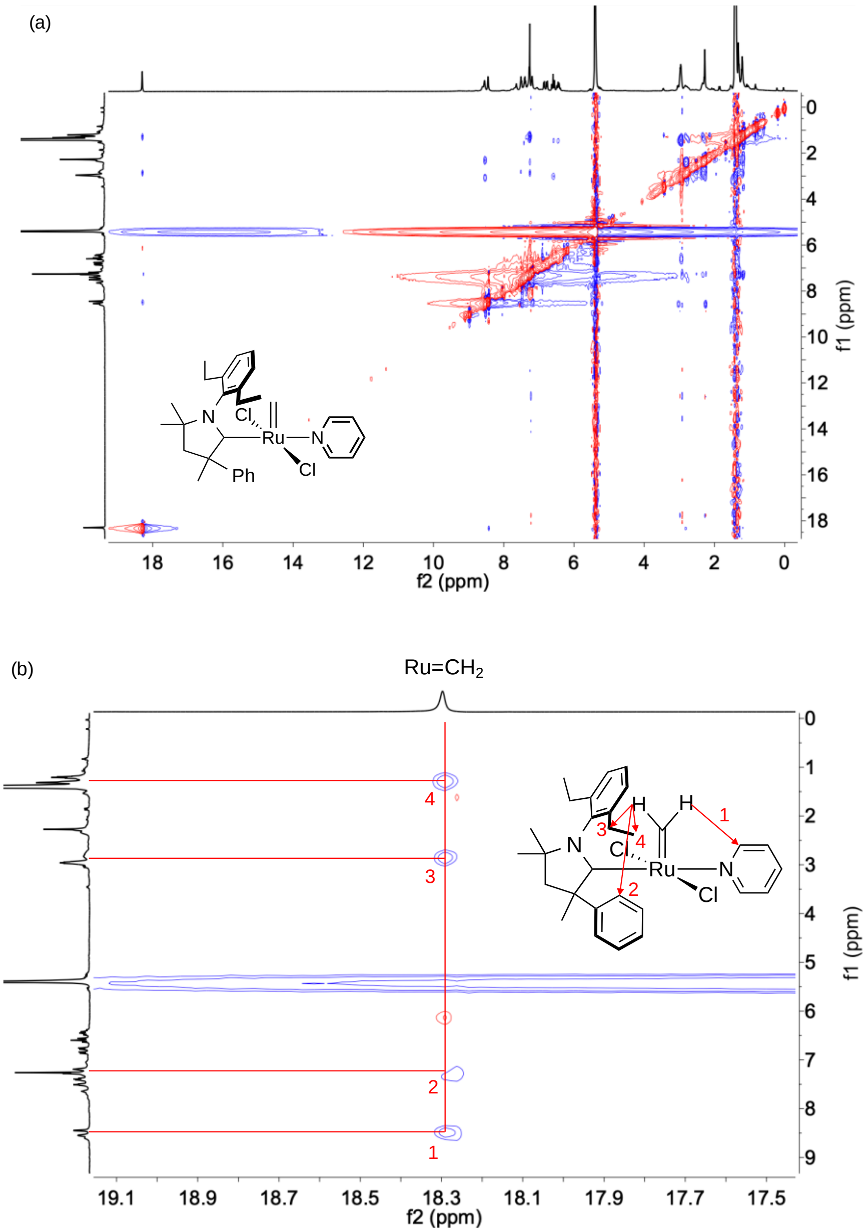

Fig. S6 ^{1}H NOESY-NMR spectra (500 MHz, $CDCl_3$, –40 °C) for in situ-generated $RuCl_2(C1^{Ph})(py)(=CH_2)$, **2-C1^{Ph}**. (a) Full NOESY spectrum. (b) Expansion, showing NOE interactions of the methylidene protons ([Ru]=CH_2) with the NAr and CPh substituents flanking both sides of the carbene carbon, consistent with the predicted electrostatic interactions for the **C1^{Ph}** ligand. (Also observed are interactions with the pyridine *o*-C*H*).

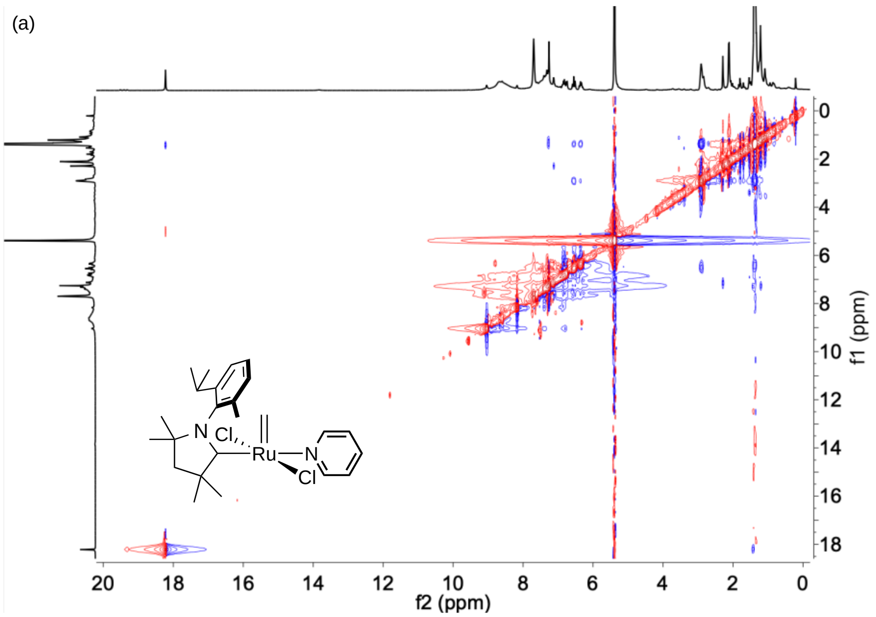

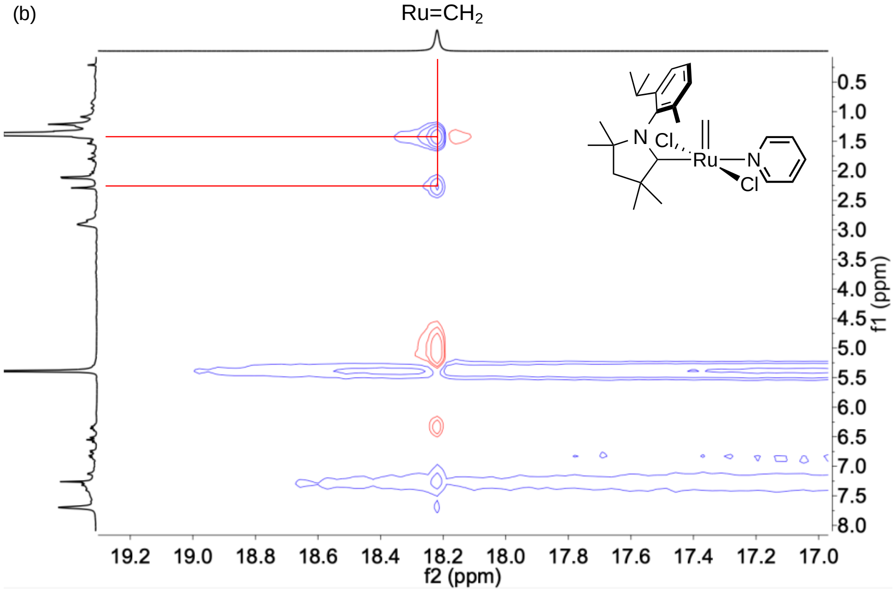

Fig. S7 ^{1}H NOESY-NMR spectra (500 MHz, $CDCl_3$, −40 °C) for in situ-generated $RuCl_2(C2^{Me})(py)(=CH_2)$, **2-C2^{Me}**. (a) Full NOESY spectrum. (b) Expansion, showing NOE interactions of the methylidene protons ([Ru]=CH_2) with two sets of methyl protons, tentatively assigned as the NAr *o*-Me and *o*-C*H*Me$_2$ groups. Confident assignment was hampered by fast bimolecular decomposition, and signal overlap with the phosphonium ylide side-product [$H_2C=CHP^iPr_3$][OTf].

2 Computational methods

2.1 Model building

The construction of molecular structures, conformational searches, and preliminary strain relaxations of molecular models were performed with Spartan18[2] using its implementation of Merck's force field (MMFF94)[3] and of the semi-empirical method PM6.[4] These calculations were usually coupled with manually set geometrical constraints in the surrounding of the metal centers to protect special geometrical features that are not well described by empirical and semiempirical methods. All density functional theory (DFT) calculations were performed with the Gaussian 16 suite of programs, versions 09 B.01[5] and 16 C.01.[6]

2.2 Geometry optimization

Geometry optimization was performed using the Gaussian 16 implementation of the generalized-gradient approximation (GGA) functional of Perdew, Burke and Ernzerhof (PBE).[7,8]

All atoms except ruthenium were described by Dunning's correlation-consistent valence double-ζ plus polarization basis sets (cc-pVDZ),[9,10] as retrieved from the EMSL basis set exchange database.[11,12] Ruthenium was described with the Stuttgart 28-electron relativistic effective core potential (ECP28MDF retrieved from the Stuttgart/Cologne group website)[13] in combination with the correlation-consistent valence double-ζ plus polarization basis set (cc-pVDZ-PP)[13] retrieved from EMSL basis set exchange database.[11,12]

The Gaussian 16 "ultrafine" grid was explicitly specified for numerical integration (keyword int=ultrafine), which implies that the "ultrafine" grid was used also for the analytical Hessian calculations. Geometries were optimized using tight convergence criteria (max. force $1.5\cdot10^{-5}$ a.u., RMS force $1.0\cdot10^{-5}$ a.u., max. force $6.0\cdot10^{-5}$ a.u., RMS force $4.0\cdot10^{-5}$ a.u.), without symmetry constraints, using the following convergence criteria for the self-consistent field (SCF) optimization procedure: RMS change in density matrix < $1.0\cdot10^{-9}$, max. change in density matrix < $1.0\cdot10^{-7}$.

Electrostatic and non-electrostatic solvation effects in chloroform (the solvent used in the experiments) were taken into account by using the polarizable continuum model (PCM) in combination with the "Dis", "Rep", and "Cav" keywords and the built-in program values (dielectric constant, number density, etc.).[14–17] The solute cavity was constructed using the united atom topological model with atomic radii optimized for Hartree−Fock (termed "UAHF").[17–20]

All stationary points were characterized by the eigenvalues of the analytically calculated Hessian matrix, confirming the absence (for minima) or presence of a single negative eigenvalue (for transition states). The translational, rotational, and vibrational components of the thermal corrections to enthalpies and Gibbs free energies were calculated within the ideal-gas, rigid-rotor, and harmonic oscillator approximations considering a temperature of 298 K, except that all frequencies below 100 cm^{-1} were shifted to 100 cm^{-1} when calculating the vibrational component of the entropy (i.e., the quasi-harmonic oscillator approximation)[21] to prevent the asymptotic behavior of the harmonic approximation with modes of very low frequencies.

Energies ($E_{PBE}^{CHCl_3}$) and thermal correction ($G_{PBE\ qh}^{CHCl_3\ 298K}$) for all molecular models are reported in Table S2. The input files of the geometry optimizations and Hessian calculations are available in the ioChem-BD repository[22,23] at https://doi.org/10.19061/iochem-bd-6-113 (embargoed data set, for reviewers: https://iochem-bd.bsc.es/browse/review-collection/100/199739/f2737266dd1e9365c07d406b).

2.3 Single-point energy calculations

All single-point energy calculations were performed with the Gaussian 16 implementation of the generalized gradient approximation (GGA)functional of Perdew, Burke and Ernzerhof (PBE)[7,8] including Grimme's D3 empirical dispersion term[24] with revised Becke-Johnson damping (overall labelled PBE-D3M(BJ) for brevity),[25] and the meta-GGA functional of Zhao and Truhlar M06L[26] including Grimme's D3 empirical dispersion term[27] (overall labelled M06L-D3). Ruthenium was described by the ECP28MDF relativistic effective core potential[13] accompanied by a correlation-consistent valence quadruple-ζ plus polarization basis set (ECP28MDF_VQZ),[13] both obtained from the Stuttgart/Cologne Group website.[28] Carbon and hydrogen atoms were described by valence quadruple-ζ plus polarization (EMSL: cc-pVQZ)[11,12] basis sets.[10] All other atoms were described by the valence quadruple-ζ plus polarization augmented with diffuse functions (EMSL: aug-cc-pVQZ),[9,11,12,29] Electrostatic and non-electrostatic

solvation effects in chloroform (the solvent used in the experiments) were taken into account by using the polarizable continuum model (PCM) in combination with the "Dis", "Rep", and "Cav" keywords and the built-in program values (dielectric constant, number density, etc.).[14–17] The solute cavity was constructed using the united atom topological model with atomic radii optimized for Hartree–Fock (termed "UAHF").[17–20] Numerical integrations were performed with the "ultrafine" grid of Gaussian 16, and the self-consistent field (SCF) density-based convergence criterion was set to 10^{-5} (RMS change in density matrix < $1.0\cdot10^{-5}$, max. change in density matrix = $1.0\cdot10^{-3}$).

Energy values with both PBE (i.e., $E^{\mathrm{CHCl_3}}_{\text{PBE-D3M(BJ)}}$) and M06L (i.e., $E^{\mathrm{CHCl_3}}_{M06L\text{-D3}}$) for all molecular models are reported in Table S2. The input files of the single point energy calculations are available in the ioChem-BD repository[22,23] at https://doi.org/10.19061/iochem-bd-6-113 (embargoed data set, for reviewers: https://iochem-bd.bsc.es/browse/review-collection/100/199739/f2737266dd1e9365c07d406b).

2.4 Calculation of Gibbs free energies

Gibbs free energies were calculated at 298 K according to equation 1, when considering the PBE-D3M(BJ) model, or equation 2, when applying the M06L-D3 model:

$$G^{\mathrm{CHCl_3}\ 298\mathrm{K}\ [1\mathrm{M}]}_{\text{PBE-D3M(BJ)}} = E^{\mathrm{CHCl_3}}_{\text{PBE-D3M(BJ)}} + G^{\mathrm{CHCl_3}\ 298\mathrm{K}}_{\text{PBE}\ qh} + G^{298\mathrm{K}}_{1\mathrm{atm}\rightarrow 1\mathrm{M}} \qquad (1)$$

$$G^{\mathrm{CHCl_3}\ 298\mathrm{K}\ [1\mathrm{M}]}_{M06L\text{-D3}} = E^{\mathrm{CHCl_3}}_{M06L\text{-D3}} + G^{\mathrm{CHCl_3}\ 298\mathrm{K}}_{\text{PBE}\ qh} + G^{298\mathrm{K}}_{1\mathrm{atm}\rightarrow 1\mathrm{M}} \qquad (2)$$

where $E^{\mathrm{CHCl_3}}_{\text{PBE-D3M(BJ)}}$ and $E^{\mathrm{CHCl_3}}_{M06L\text{-D3}}$ are the potential energy resulting from single-point calculations with PBE-D3M(BJ) and M06L-D3, respectively, and include the contributions from the implicit solvation model; $G^{\mathrm{CHCl_3}\ 298\mathrm{K}}_{\text{PBE}\ qh}$ is the thermal correction to the Gibbs free energy calculated at the geometry optimization level with the quasi-harmonic approximation at 298 K; and $G^{298\mathrm{K}}_{1\mathrm{atm}\rightarrow 1\mathrm{M}}$ is the standard state correction corresponding to 1 M solution (but exhibiting infinite-dilution, ideal-gas-like behavior), which is equal to 1.89 kcal mol^{-1} (= RT·ln(24.46)) at room temperature. Table S2 reports Gibbs free energy values calculated with both PBE-D3M(BJ) and M06L-D3 methods ($G^{\mathrm{CHCl_3}\ 298\mathrm{K}\ [1\mathrm{M}]}_{\text{PBE-D3M(BJ)}}$ and $G^{\mathrm{CHCl_3}\ 298\mathrm{K}\ [1\mathrm{M}]}_{M06L\text{-D3}}$) and the relative values ($\Delta G^{\mathrm{CHCl_3}\ 298\mathrm{K}\ [1\mathrm{M}]}_{\text{PBE-D3M(BJ)}}$ and $\Delta G^{\mathrm{CHCl_3}\ 298\mathrm{K}\ [1\mathrm{M}]}_{M06L\text{-D3}}$) calculated with respect to the metallacyclobutane **4** with a given carbene ligand. Namely, molecular models **M20**, **M21**, or **M22** (see Table S2) are the reference points for each species bearing the H_2IMes, **C1$^{\mathbf{Ph}}$**, or **C2$^{\mathbf{Me}}$** ligand, respectively.

2.5 Natural bond orbital (NBO) analyses

The natural bond orbital analyses were performed with the NBO7 software,[30] using the electron density of the single-point energy calculations as input.

The ligand–metal σ-donation of the carbene ligands (H_2IMes, **C1$^{\mathbf{Ph}}$**, and **C2$^{\mathbf{Me}}$**) in metallacyclobutane complex **4** was determined as the difference between the electron population of the carbene lone pair (LP) in **4** and that of the corresponding carbene ligand 'frozen' in the geometry of **4**. Similarly, the π-back-donation to each of the carbene ligands in **4** was determined as the difference between the electron population of the carbene lone vacancy (LV) in **4** and that of the corresponding carbene ligand 'frozen' in the geometry of **4**. To ensure a comparable set of orbitals between the complexes and the 'frozen' ligand fragments, the Lewis structures were explicitly required (via the $CHOOSE input section) to have a lone pair (LP) at the carbene carbon atom as well as on the adjacent N-atom.

Bond orders in the transition states for ethylene and styrene metathesis were estimated using the Wiberg bond index (WBI).[31]

The steric exchange repulsion energy between the carbene, the methylidene, and the chloride ligands in methylidene complexes **2** and **2'** was calculated using natural steric analysis.[32] This was accomplished by summing all pairwise steric energies contributions ≥ 0.1 kcal/mol. The methylidene moiety employed in the calculations included the ruthenium–carbon σ- and π-bonds and the C–H bonds, whereas the lone pairs and the ruthenium–chloride σ-bonds were included for the chloride ligands.

To ensure a comparable set of orbitals, the second-order perturbation theory analysis of transition state **TS4-5** (Table S1) was performed with the Lewis structures were explicitly required (via the $CHOOSE input section) to

have a lone pair (LP) at the carbene carbon atom as well as on the adjacent N-atom as shown in Table S1, and a single covalent bond at the braking β-C–H interaction.

2.6 Calculated steric volumes

Buried volumes (the percentage of a sphere that is occupied, $\%V_{bur}$) and steric maps for the carbene ligand H_2IMes, **C1^{Ph}**, and **C2^{Me}** in the DFT-optimized metallacyclobutane **4** were obtained using the SambVca 2.1 web application developed by Cavallo and co-workers.[33] A sphere of radius 3.5 Å centered on ruthenium was used, and the van der Waals radii were those of Bondi,[34] scaled by 1.17. The mesh spacing for numerical integration was 0.10 Å. Hydrogen atoms were excluded.

3 Computational results

3.1 Molecular geometries

A data set collection of computational results including all molecular geometries and energies is available in the ioChem-BD repository[22,23] at https://doi.org/10.19061/iochem-bd-6-113 (embargoed data set, for reviewers: https://iochem-bd.bsc.es/browse/review-collection/100/199739/f2737266dd1e9365c07d406b).

3.2 Natural bond orbital (NBO) analyses

Calculated Wiberg bond indices (WBIs) of the transition states for ethylene and styrene metathesis are shown in Fig. S8. As discussed in the main part of the paper, these indices, or bond orders, suggest that the transition states for ethylene metathesis (**TS3'-4/TS4-3**, deemed 'early' transition states) resemble π-complexes more than metallacyclobutane intermediates. Conversely, the 'late' transition states for self-metathesis of styrene (a model 1-alkene) are more like metallacyclobutane intermediates than π-complexes.

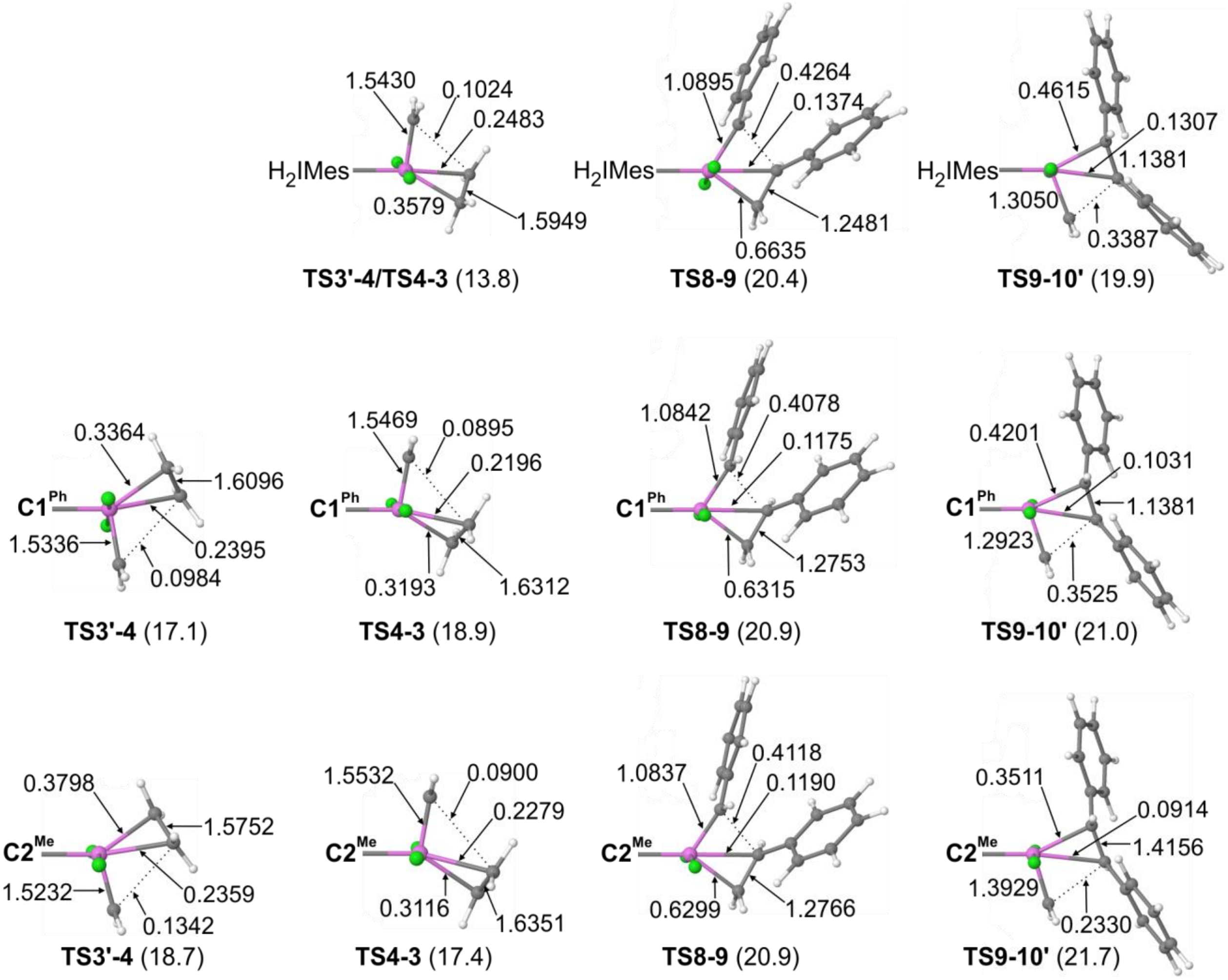

Fig. S8 Selected Wiberg bond indices (WBIs) for the key transition states of ethylene and 1-alkene metathesis.

The NBO-based second-order perturbation analysis of donor–acceptor interactions in **TS4-5** (Table S1) confirms that the carbene lone pair and the β-C–H bond compete for σ-donation to the same ruthenium acceptor orbital. Simultaneously, the corresponding carbene π-acceptor orbital and the antibonding β-C–H and Ru–C orbitals compete for the same Ru lone pair.

Table S1 Selected interaction energies E(2) from second-order perturbation theory analysis of the Fock matrix in NBO basis.[a]

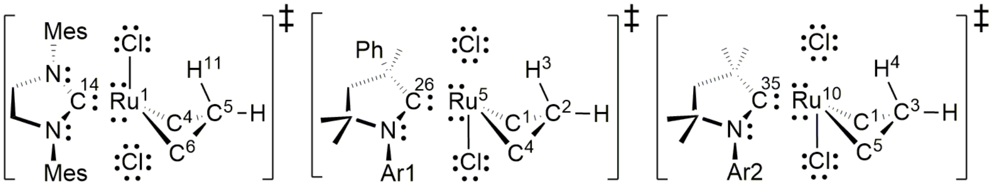

TS4-5-H$_2$IMes **TS4-5-C1^{Ph}** **TS4-5-C2^{Me}**

Mes = mesityl, Ar1 = 2,6-diethylphenyl, Ar2 = 2-isopropyl-6-methylphenyl

Complex	Donor NBO	Acceptor NBO	E(2) (kcal/mol)
**TS4-5-**H$_2$IMes (**M31**)	LP (1) C 14	LV (1)Ru 1	141.25
	LP (1) C 14	BD*(1)Ru 1- C 4	22.48
	BD (1) C 5- H 11	LV (1)Ru 1	50.35
	BD (1)Ru 1- C 4	LV (1)Ru 1	5.30
	BD (1)Ru 1- C 6	LV (1)Ru 1	8.82
	BD (1) C 4- C 5	LV (1)Ru 1	7.64
	BD (1) C 5- C 6	LV (1)Ru 1	8.44
	LP (2)Ru 1	LV (1) C 14	37.80
	LP (2)Ru 1	BD*(1)Ru 1- C 4	6.99
	LP (2)Ru 1	BD*(1)Ru 1- C 6	6.56
	LP (2)Ru 1	BD*(1) C 5- H 11	6.86
**TS4-5-C1^{Ph}** (**M33**)	LP (1) C 26	LV (1)Ru 5	143.18
	LP (1) C 26	BD*(1) C 4-Ru 5	22.21
	BD (1) C 1- C 2	LV (1)Ru 5	7.13
	BD (1) C 1-Ru 5	LV (1)Ru 5	11.06
	BD (1) C 2- H 3	LV (1)Ru 5	61.94
	BD (1) C 2- C 4	LV (1)Ru 5	7.07
	BD (1) C 4-Ru 5	LV (1)Ru 5	12.13
	LP (2)Ru 5	LV (1) C 26	19.62
	LP (2)Ru 5	BD*(1) C 1-Ru 5	6.45
	LP (2)Ru 5	BD*(1) C 2- H 3	7.57
	LP (2)Ru 5	BD*(1) C 4-Ru 5	8.78
**TS4-5-C2^{Me}** (**M37**)	LP (1) C 35	LV (1)Ru 10	135.15
	LP (1) C 35	BD*(1) C 5-Ru 10	19.53
	BD (1) C 3- H 4	LV (1)Ru 10	63.71
	BD (1) C 1- C 3	LV (1)Ru 10	7.19
	BD (1) C 1-Ru 10	LV (1)Ru 10	9.39
	BD (1) C 3- C 5	LV (1)Ru 10	6.75
	BD (1) C 5-Ru 10	LV (1)Ru 10	10.26

	LP (2)Ru 10	LV (1) C 35	17.34
	LP (2)Ru 10	BD*(1) C 1-Ru 10	8.96
	LP (2)Ru 10	BD*(1) C 3- H 4	10.00
	LP (2)Ru 10	BD*(1) C 5-Ru 10	11.06
	LP (2)Ru 10	BD*(1)Ru 10-Cl 12	12.47

[a] Interaction energies <5 kcal/mol not shown.

3.3 Calculated steric volumes

Steric maps and $\%V_{bur}$ describe the steric effects of the carbene ligands at the Ru center and give an idea of the steric potential of the ligand, but do not quantify the steric interaction with other ligands.

The steric properties of the H_2IMes, **C1^{Ph}**, and **C2^{Me}** complexes were expected to differ based on the nature of the substituents flanking the carbene carbon, and the shorter bonds to Ru for the CAAC complexes vs H_2IMes. However, the calculated percent buried volumes[35] ($\%V_{bur}$; Fig. 9) of **4** bearing these three carbenes differ by only 2.6 percentage points. The steric distinction between the three carbenes thus appears to be small, albeit **C1^{Ph}** is predicted to be slightly more sterically demanding. On imposing an identical Ru–carbene bond distance of 1.958 Å (a value determined from calculations on the MCB for **C2^{Me}**), $\%V_{bur}$ for **C1^{Ph}** remained slightly higher, whereas H_2IMes and **C2^{Me}** were comparable (36.4, vs 34.9 or 34.3, respectively).

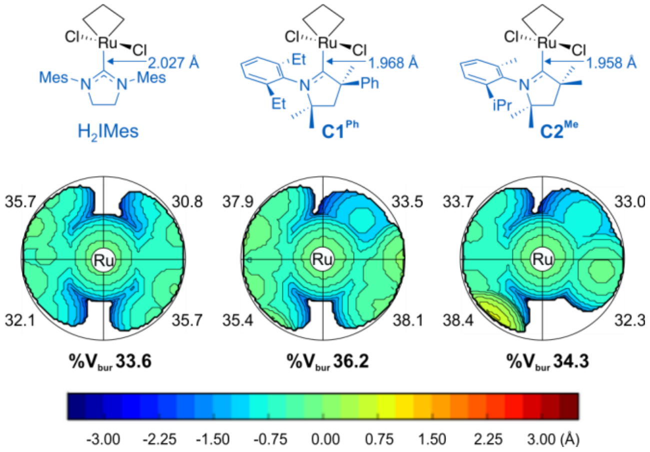

Fig. S9 Steric maps[35] of the metallacyclobutane **4** for the H_2IMes, **C1^{Ph}** , and **C2^{Me}** species.

3.4 Calculated total and free energies

Table S2 Calculated energies and standard-state Gibbs free energies.

Species	Carbene ligand	Molecular model ID	Spin mulplicity	$E^{CHCl_3}_{PBE}$ [a.u.]	$G^{CHCl_3\,298K}_{PBE\ qh}$ [a.u.]	$E^{CHCl_3}_{PBE\text{-}D3M(BJ)}$ [a.u.]	$E^{CHCl_3}_{M06L\text{-}D3}$ [a.u.]	$G^{CHCl_3\,298K\,[1M]}_{PBE\text{-}D3M(BJ)}$ [a.u.]	$G^{CHCl_3\,298K\,[1M]}_{M06L\text{-}D3}$ [a.u.]	$\Delta G^{CHCl_3\,298K\,[1M]}_{PBE\text{-}D3M(BJ)}$ [kcal/mol][a]	$\Delta G^{CHCl_3\,298K\,[1M]}_{M06L\text{-}D3}$ [kcal/mol][a]
2-Isopropoxy-5-nitrostyrene	-	2-Isopropoxy-5-nitrostyrene	1	-706.533564	0.175338	-706.832155	-707.535013	-706.653800	-707.356658	-	-
ethylene	-	ethylene	1	-78.470361	0.028259	-78.507666	-78.607297	-78.476390	-78.576021	-	-
styrene	-	styrene	1	-309.256296	0.097909	-309.384010	-309.730351	-309.283085	-309.629425	-	-
trans-stilbene	-	trans-stilbene	1	-540.041871	0.171082	-540.260654	-540.853663	-540.086556	-540.679564	-	-
H_2IMes	-	**M1**	1	-924.221956	0.351671	-924.620247	-925.637232	-924.265560	-925.282544	-	-
**C1^{Ph}**	-	**M2**	1	-947.397726	0.396237	-947.817443	-948.880232	-947.418189	-948.480978	-	-
**C2^{Me}**	-	**M3**	1	-755.889242	0.346848	-756.234216	-757.098000	-755.884350	-756.748135	-	-
**1**	H_2IMes	**M4**	1	-2606.346257	0.528255	-2607.120647	-2609.136929	-2606.589375	-2608.605658	5.7	2.7
**1**	**C1^{Ph}**	**M5**	1	-2629.532651	0.574981	-2630.330547	-2632.392598	-2629.752549	-2631.814600	3.9	2.7
**1**	**C2^{Me}**	**M6**	1	-2438.028342	0.524576	-2438.748205	-2440.612044	-2438.220612	-2440.084452	3.3	1.5
**1’**	**C1^{Ph}**	**M7**	1	-2629.533320	0.575108	-2630.329678	-2632.391941	-2629.751553	-2631.813817	4.5	3.2
**1’**	**C2^{Me}**	**M8**	1	-2438.019570	0.523433	-2438.737183	-2440.600970	-2438.210733	-2440.074520	9.5	7.8
**2'** (same as **2**)	H_2IMes	**M9**	1	-1978.271174	0.372276	-1978.772658	-1980.185210	-1978.397365	-1979.809917	14.9	12.1
**2'**	**C1^{Ph}**	**M10**	1	-2001.458188	0.418839	-2001.981356	-2003.439172	-2001.559500	-2003.017316	13.7	13.1
**2'**	**C2^{Me}**	**M11**	1	-1809.950266	0.369796	-1810.397625	-1811.658027	-1810.024812	-1811.285214	14.8	13.2
**3'** (same as **3**)	H_2IMes	**M12**	1	-2056.763425	0.423224	-2057.305620	-2058.813719	-2056.879379	-2058.387478	11.4	11.2

**3'**	**C1^{Ph}**	**M13**	1	-2079.940805	0.469633	-2080.506235	-2082.063577	-2080.033586	-2081.590928	15.2	14.6
**3'**	**C2^{Me}**	**M14**	1	-1888.433231	0.420113	-1888.921492	-1890.279964	-1888.498362	-1889.856834	16.6	16.0
**TS3'-4** (same as **TS4-3**)	H_2IMes	**M15**	1	-2056.759750	0.423221	-2057.301731	-2058.809873	-2056.875494	-2058.383636	13.8	13.6
**TS3'-4**	**C1^{Ph}**	**M16**	1	-2079.937818	0.469140	-2080.502679	-2082.059595	-2080.030522	-2081.587438	17.1	16.8
**TS3'-4**	**C1^{Ph}**	**M17**	1	-2079.938059	0.469460	-2080.502682	-2082.059810	-2080.030205	-2081.587334	17.3	16.9
**TS3'-4**	**C2^{Me}**	**M18**	1	-1888.429386	0.419924	-1888.916931	-1890.275487	-1888.493990	-1889.852546	19.3	18.6
**TS3'-4**	**C2^{Me}**	**M19**	1	-1888.430082	0.419886	-1888.917941	-1890.276538	-1888.495038	-1889.853635	18.7	18.0
**4**	H_2IMes	**M20**	1	-2056.786419	0.426175	-2057.326665	-2058.834466	-2056.897474	-2058.405275	0.0	0.0
**4**	**C1^{Ph}**	**M21**	1	-2079.970458	0.472717	-2080.533502	-2082.090001	-2080.057768	-2081.614268	0.0	0.0
**4**	**C2^{Me}**	**M22**	1	-1888.464039	0.422623	-1888.950448	-1890.307903	-1888.524808	-1889.882263	0.0	0.0
**TS4-3**	**C1^{Ph}**	**M23**	1	-2079.933830	0.468950	-2080.498738	-2082.054131	-2080.026772	-2081.582165	19.5	20.1
**TS4-3**	**C1^{Ph}**	**M24**	1	-2079.933951	0.468316	-2080.499033	-2082.055129	-2080.027701	-2081.583797	18.9	19.1
**TS4-3**	**C2^{Me}**	**M25**	1	-1888.430815	0.418832	-1888.918981	-1890.277233	-1888.497131	-1889.855384	17.4	16.9
**TS4-3**	**C2^{Me}**	**M26**	1	-1888.431007	0.419308	-1888.918968	-1890.276927	-1888.496643	-1889.854602	17.7	17.4
**3**	**C1^{Ph}**	**M27**	1	-2079.936828	0.468416	-2080.502244	-2082.058172	-2080.030811	-2081.586740	16.9	17.3
**3**	**C2^{Me}**	**M28**	1	-1888.433844	0.419280	-1888.922597	-1890.281146	-1888.500300	-1889.858850	15.4	14.7
**2**	**C1^{Ph}**	**M29**	1	-2001.456600	0.418655	-2001.982424	-2003.440260	-2001.560752	-2003.018588	12.9	12.3
**2**	**C2^{Me}**	**M30**	1	-1809.954357	0.368612	-1810.402312	-1811.661629	-1810.030684	-1811.290001	11.1	10.2
**TS4-5**	H_2IMes	**M31**	1	-2056.740144	0.423919	-2057.281853	-2058.795454	-2056.854918	-2058.368519	26.7	23.1
**TS4-5** (Pathway A)	**C1^{Ph}**	**M32**	1	-2079.914804	0.469265	-2080.478198	-2082.034562	-2080.005916	-2081.562280	32.5	32.6
**TS4-5** (Pathway B)	**C1^{Ph}**	**M33**	1	-2079.915353	0.468701	-2080.478777	-2082.035802	-2080.007059	-2081.564085	31.8	31.5
**TS4-5** (Pathway C)	**C1^{Ph}**	**M34**	1	-2079.913581	0.468128	-2080.475974	-2082.032467	-2080.004830	-2081.561322	33.2	33.2
**TS4-5** (Pathway D)	**C1^{Ph}**	**M35**	1	-2079.915488	0.468250	-2080.477942	-2082.032977	-2080.006675	-2081.561709	32.1	33.0

**TS4-5** (Pathway A)	**C2^{Me}**	**M36**	1	-1888.412557	0.419860	-1888.898427	-1890.256774	-1888.475551	-1889.833897	30.9	30.3
**TS4-5** (Pathway B)	**C2^{Me}**	**M37**	1	-1888.413076	0.419847	-1888.899187	-1890.258364	-1888.476324	-1889.835501	30.4	29.3
**TS4-5** (Pathway C)	**C2^{Me}**	**M38**	1	-1888.410279	0.418237	-1888.896119	-1890.254562	-1888.474866	-1889.833308	31.3	30.7
**TS4-5** (Pathway D)	**C2^{Me}**	**M39**	1	-1888.407865	0.418085	-1888.893517	-1890.248648	-1888.472415	-1889.827546	32.9	34.3
**5**	H_2IMes	**M40**	1	-2056.786254	0.424091	-2057.326562	-2058.832694	-2056.899455	-2058.405587	-1.2	-0.2
**5** (Pathway A)	**C1^{Ph}**	**M41**	1	-2079.961974	0.469813	-2080.525056	-2082.077110	-2080.052227	-2081.604281	3.5	6.3
**5** (Pathway B)	**C1^{Ph}**	**M42**	1	-2079.960418	0.469236	-2080.522872	-2082.074139	-2080.050620	-2081.601887	4.5	7.8
**5** (Pathway C)	**C1^{Ph}**	**M43**	1	-2079.963509	0.470694	-2080.525382	-2082.076787	-2080.051671	-2081.603076	3.8	7.0
**5** (Pathway D)	**C1^{Ph}**	**M44**	1	-2079.961502	0.469718	-2080.523436	-2082.074824	-2080.050701	-2081.602090	4.4	7.6
**5** (Pathway A)	**C2^{Me}**	**M45**	1	-1888.462323	0.420510	-1888.948514	-1890.303191	-1888.524987	-1889.879664	-0.1	1.6
**5** (Pathway B)	**C2^{Me}**	**M46**	1	-1888.461547	0.420477	-1888.947152	-1890.301024	-1888.523658	-1889.877530	0.7	3.0
**5** (Pathway C)	**C2^{Me}**	**M47**	1	-1888.454430	0.420455	-1888.940239	-1890.294641	-1888.516768	-1889.871169	5.0	7.0
**5** (Pathway D)	**C2^{Me}**	**M48**	1	-1888.455968	0.419822	-1888.941451	-1890.296295	-1888.518612	-1889.873456	3.9	5.5
**TS5-6**	H_2IMes	**M49**	1	-2056.776135	0.421916	-2057.315167	-2058.821743	-2056.890234	-2058.396811	4.5	5.3
**TS5-6** (Pathway A)	**C1^{Ph}**	**M50**	1	-2079.952639	0.468335	-2080.513701	-2082.066885	-2080.042350	-2081.595534	9.7	11.8
**TS5-6** (Pathway B)	**C1^{Ph}**	**M51**	1	-2079.954287	0.467952	-2080.515770	-2082.068384	-2080.044801	-2081.597415	8.1	10.6
**TS5-6** (Pathway C)	**C1^{Ph}**	**M52**	1	-2079.956138	0.467959	-2080.518967	-2082.072505	-2080.047991	-2081.601529	6.1	8.0
**TS5-6** (Pathway D)	**C1^{Ph}**	**M53**	1	-2079.951137	0.468123	-2080.513399	-2082.067661	-2080.042260	-2081.596522	9.7	11.1
**TS5-6** (Pathway A)	**C2^{Me}**	**M54**	1	-1888.447795	0.418051	-1888.932780	-1890.288621	-1888.511712	-1889.867553	8.2	9.2
**TS5-6** (Pathway B)	**C2^{Me}**	**M55**	1	-1888.447894	0.418360	-1888.932511	-1890.288382	-1888.511134	-1889.867006	8.6	9.6

**TS5-6** (Pathway C)	**C2^{Me}**	**M56**	1	-1888.450099	0.418727	-1888.935118	-1890.290415	-1888.513374	-1889.868671	7.2	8.5
**TS5-6** (Pathway D)	**C2^{Me}**	**M57**	1	-1888.449602	0.418363	-1888.934243	-1890.288952	-1888.512863	-1889.867572	7.5	9.2
**6**	H_2IMes	**M58**	1	-2056.799619	0.426378	-2057.340548	-2058.855047	-2056.911153	-2058.425652	-8.6	-12.8
**6** (Pathway A)	**C1^{Ph}**	**M59**	1	-2079.983790	0.471174	-2080.553427	-2082.105843	-2080.079236	-2081.631652	-13.5	-10.9
**6** (Pathway B)	**C1^{Ph}**	**M60**	1	-2079.989038	0.472074	-2080.558579	-2082.112259	-2080.083488	-2081.637169	-16.1	-14.4
**6** (Pathway C)	**C1^{Ph}**	**M61**	1	-2079.981923	0.471786	-2080.551649	-2082.104318	-2080.076847	-2081.629515	-12.0	-9.6
**6** (Pathway D)	**C1^{Ph}**	**M62**	1	-2079.983542	0.472048	-2080.553123	-2082.105606	-2080.078058	-2081.630541	-12.7	-10.2
**6** (Pathway A)	**C2^{Me}**	**M63**	1	-1888.479531	0.422506	-1888.972326	-1890.328292	-1888.546804	-1889.902770	-13.8	-12.9
**6** (Pathway B)	**C2^{Me}**	**M64**	1	-1888.477901	0.423663	-1888.970054	-1890.325176	-1888.543375	-1889.898497	-11.7	-10.2
**6** (Pathway C)	**C2^{Me}**	**M65**	1	-1888.479014	0.422459	-1888.971614	-1890.326117	-1888.546139	-1889.900642	-13.4	-11.5
**6** (Pathway D)	**C2^{Me}**	**M66**	1	-1888.482298	0.422411	-1888.975370	-1890.330372	-1888.549943	-1889.904944	-15.8	-14.2
**TS8-9**	H_2IMes	**M67**	1	-2518.316473	0.572230	-2519.053575	-2521.052887	-2518.478328	-2520.477640	20.4	21.6
**TS8’-9** (Pathway 1)	**C1^{Ph}**	**M68**	1	-2541.499072	0.618590	-2542.256948	-2544.303045	-2541.635342	-2543.681438	22.5	24.9
**TS8’-9** (Pathway 2)	**C1^{Ph}**	**M69**	1	-2541.499834	0.618026	-2542.256489	-2544.302077	-2541.635447	-2543.681035	22.4	25.1
**TS8-9** (Pathway 3)	**C1^{Ph}**	**M70**	1	-2541.495673	0.616884	-2542.256880	-2544.302691	-2541.636980	-2543.682791	21.4	24.0
**TS8-9** (Pathway 4)	**C1^{Ph}**	**M71**	1	-2541.497904	0.617038	-2542.257941	-2544.304576	-2541.637887	-2543.684522	20.9	22.9
**TS8’-9** (Pathway 1)	**C2^{Me}**	**M72**	1	-2349.993847	0.568072	-2350.673392	-2352.520367	-2350.102303	-2351.949278	22.5	25.0
**TS8’-9** (Pathway 2)	**C2^{Me}**	**M73**	1	-2349.993811	0.567172	-2350.673105	-2352.520181	-2350.102916	-2351.949992	22.1	24.5
**TS8-9** (Pathway 3)	**C2^{Me}**	**M74**	1	-2349.991108	0.568115	-2350.671978	-2352.519508	-2350.100846	-2351.948377	23.4	25.5
**TS8-9** (Pathway 4)	**C2^{Me}**	**M75**	1	-2349.992790	0.568132	-2350.676064	-2352.524316	-2350.104915	-2351.953167	20.9	22.5

**TS9-10**	H_2IMes	**M76**	1	-2518.314820	0.568935	-2519.051119	-2521.050955	-2518.479167	-2520.479003	19.9	20.8
**TS9-10** (Pathway 1)	**C1^{Ph}**	**M77**	1	-2541.495764	0.614867	-2542.252954	-2544.299354	-2541.635070	-2543.681471	22.6	24.9
**TS9-10** (Pathway 2)	**C1^{Ph}**	**M78**	1	-2541.494656	0.615254	-2542.253150	-2544.300398	-2541.634880	-2543.682128	22.8	24.4
**TS9-10'** (Pathway 3)	**C1^{Ph}**	**M79**	1	-2541.497651	0.615820	-2542.255967	-2544.302342	-2541.637130	-2543.683505	21.4	23.6
**TS9-10'** (Pathway 4)	**C1^{Ph}**	**M80**	1	-2541.497268	0.614968	-2542.255731	-2544.303564	-2541.637746	-2543.685580	21.0	22.3
**TS9-10** (Pathway 1)	**C2^{Me}**	**M81**	1	-2349.990048	0.565741	-2350.668934	-2352.516204	-2350.100176	-2351.947447	23.9	26.1
**TS9-10** (Pathway 2)	**C2^{Me}**	**M82**	1	-2349.989007	0.564971	-2350.668213	-2352.515461	-2350.100225	-2351.947474	23.8	26.1
**TS9-10'** (Pathway 3)	**C2^{Me}**	**M83**	1	-2349.988647	0.565913	-2350.671333	-2352.522256	-2350.102403	-2351.953326	22.5	22.4
**TS9-10'** (Pathway 4)	**C2^{Me}**	**M84**	1	-2349.988834	0.565845	-2350.672414	-2352.520541	-2350.103552	-2351.951679	21.7	23.5
**TS2'-2**	**C1^{Ph}**	**M85**	1	-2001.442601	0.419180	-2001.967274	-2003.425039	-2001.545077	-2003.002842	22.8	22.2
**TS2'-2**	**C2^{Me}**	**M86**	1	-1809.927805	0.371310	-1810.373940	-1811.631766	-1809.999613	-1811.257439	30.6	30.6
**TS3'-3**	**C2^{Me}**	**M87**	1	-1888.391556	0.419533	-1888.879370	-1890.238459	-1888.456821	-1889.815909	42.7	41.6
$RuCl_2(CH_2)_3$ fragment	**C1^{Ph}**	**M88**	3	-1132.492216	0.044638	-1132.619037	-1133.122870	-1132.571382	-1133.075216	-	-

[a] The relative Gibbs free energy of each species bearing the H_2IMes, **C1^{Ph}**, or **C2^{Me}** ligand is calculated with respect to the corresponding energy model of metallacyclobutane **4**, namely, **M20**, **M21**, or **M22** respectively.

4 References

1 Nascimento, D. L., Foscato, M., Occhipinti, G., Jensen, V. R. and Fogg, D. E., *J. Am. Chem. Soc.*, 2021, **143**, 11072–11079.
2 *Spartan'18*, Wavefunction, Inc., Irvine, CA, 2018.
3 T. A. Halgren, *J. Comput. Chem.*, 1996, **17**, 490–519.
4 J. J. P. Stewart, *J. Mol. Model.*, 2007, **13**, 1173–1213.
5 M. J. Frisch, G. W. Trucks, H. B. Schlegel, G. E. Scuseria, M. A. Robb, J. R. Cheeseman, G. Scalmani, V. Barone, G. A. Petersson, H. Nakatsuji, X. Li, M. Caricato, A. V. Marenich, J. Bloino, B. G. Janesko, R. Gomperts, B. Mennucci, H. P. Hratchian, J. V. Ortiz, A. F. Izmaylov, J. L. Sonnenberg, D. Williams-Young, F. Ding, F. Lipparini, F. Egidi, J. Goings, B. Peng, A. Petrone, T. Henderson, D. Ranasinghe, V. G. Zakrzewski, J. Gao, N. Rega, G. Zheng, W. Liang, M. Hada, M. Ehara, K. Toyota, R. Fukuda, J. Hasegawa, M. Ishida, T. Nakajima, Y. Honda, O. Kitao, H. Nakai, T. Vreven, K. Throssell, J. A. Jr. Montgomery, J. E. Peralta, F. Ogliaro, M. J. Bearpark, J. J. Heyd, E. N. Brothers, K. N. Kudin, V. N. Staroverov, T. A. Keith, R. Kobayashi, J. Normand, K. Raghavachari, A. P. Rendell, J. C. Burant, S. S. Iyengar, J. Tomasi, M. Cossi, J. M. Millam, M. Klene, C. Adamo, R. Cammi, J. W. Ochterski, R. L. Martin, K. Morokuma, O. Farkas, J. B. Foresman and D. J. Fox, *Gaussian 16 Revision B.01*, Wallingford CT, 2016.
6 M. J. Frisch, G. W. Trucks, H. B. Schlegel, G. E. Scuseria, M. A. Robb, J. R. Cheeseman, G. Scalmani, V. Barone, G. A. Petersson, H. Nakatsuji, X. Li, M. Caricato, A. V. Marenich, J. Bloino, B. G. Janesko, R. Gomperts, B. Mennucci, H. P. Hratchian, J. V. Ortiz, A. F. Izmaylov, J. L. Sonnenberg, D. Williams-Young, F. Ding, F. Lipparini, F. Egidi, J. Goings, B. Peng, A. Petrone, T. Henderson, D. Ranasinghe, V. G. Zakrzewski, J. Gao, N. Rega, G. Zheng, W. Liang, M. Hada, M. Ehara, K. Toyota, R. Fukuda, J. Hasegawa, M. Ishida, T. Nakajima, Y. Honda, O. Kitao, H. Nakai, T. Vreven, K. Throssell, J. A. Jr. Montgomery, J. E. Peralta, F. Ogliaro, M. J. Bearpark, J. J. Heyd, E. N. Brothers, K. N. Kudin, V. N. Staroverov, T. A. Keith, R. Kobayashi, J. Normand, K. Raghavachari, A. P. Rendell, J. C. Burant, S. S. Iyengar, J. Tomasi, M. Cossi, J. M. Millam, M. Klene, C. Adamo, R. Cammi, J. W. Ochterski, R. L. Martin, K. Morokuma, O. Farkas, J. B. Foresman and D. J. Fox, *Gaussian 16 Revision C.01*, Wallingford CT, 2016.
7 J. Perdew, K. Burke and M. Ernzerhof, *Phys. Rev. Lett.*, 1997, **78**, 1396.
8 J. Perdew, K. Burke and M. Ernzerhof, *Phys. Rev. Lett.*, 1996, **77**, 3865–3868.
9 T. H. Dunning, *J. Chem. Phys.*, 1989, **90**, 1007–1023.
10 D. E. Woon and T. H. Dunning, *J. Chem. Phys.*, 1993, **98**, 1358–1371.
11 D. Feller, *J. Comput. Chem.*, 1996, **17**, 1571–1586.
12 K. L. Schuchardt, B. T. Didier, T. Elsethagen, L. S. Sun, V. Gurumoorthi, J. Chase, J. Li and T. L. Windus, *J. Chem. Inf. Model.*, 2007, **47**, 1045–1052.
13 K. A. Peterson, D. Figgen, M. Dolg and H. Stoll, *J. Chem. Phys.*, 2007, **126**, 124101.
14 M. Cossi, G. Scalmani, N. Rega and V. Barone, *J. Chem. Phys.*, 2002, **117**, 43–54.
15 G. Scalmani and M. J. Frisch, 2010, **132**, 114110.
16 J. Tomasi, B. Mennucci and R. Cammi, *Chem. Rev.*, 2005, **105**, 2999–3093.
17 J. Tomasi and M. Persico, *Chem Rev*, 1994, **94**, 2027–2094.
18 J. Tomasi, *Theor. Chem. Acc.*, 2004, **112**, 184–203.
19 C. J. Cramer and D. G. Truhlar, *Phys. Chem. Chem. Phys.*, 2009, **11**, 10757–10816.
20 A. Klamt, B. Mennucci, J. Tomasi, V. Barone, C. Curutchet, M. Orozco and F. J. Luque, *Acc. Chem. Res.*, 2009, **42**, 489–492.
21 R. F. Ribeiro, A. V. Marenich, C. J. Cramer and D. G. Truhlar, *J. Phys. Chem. B*, 2011, **115**, 14556–14562.
22 M. Álvarez-Moreno, C. de Graaf, N. López, F. Maseras, J. M. Poblet and C. Bo, *J. Chem. Inf. Model.*, 2015, **55**, 95–103.
23 C. Bo, F. Maseras and N. López, *Nat. Catal.*, 2018, **1**, 809–810.
24 S. Grimme, S. Ehrlich and L. Goerigk, *J. Comput. Chem.*, 2011, **32**, 1456–1465.
25 D. G. A. Smith, L. A. Burns, K. Patkowski and C. D. Sherrill, *J. Phys. Chem. Lett.*, 2016, **7**, 2197–2203.
26 Z. Yan and D. G. Truhlar, *J. Chem. Phys.*, 2006, **125**, 194101-1–18.
27 S. Grimme, J. Antony, S. Ehrlich and H. Krieg, *J. Chem. Phys.*, 2010, **132**, 154104.
28 Energy-consistent Pseudopotentials of the Stuttgart/Cologne Group, http://www.tc.uni-koeln.de/PP/clickpse.en.html.
29 R. A. Kendall, T. H. Dunning and R. J. Harrison, *J Chem Phys*, 1992, **96**, 6796–6806.
30 E. D. Glendening, J. K. Badenhoop, A. E. Reed, J. E. Carpenter, J. A. Bohmann, C. M. Morales, P. Karafiloglou, C. R. Landis and F. Weinhold, *NBO 7.0*, Theoretical Chemistry Institute, University of Wisconsin, Madison, WI, USA, 2018.
31 K. B. Wiberg, *Tetrahedron*, 1968, **24**, 1083-.
32 J. K. Badenhoop and F. Weinhold, *J. Chem. Phys.*, 1997, **107**, 5422–5432.
33 A. Poater, B. Cosenza, A. Correa, S. Giudice, F. Ragone, V. Scarano and L. Cavallo, *Eur. J. Inorg. Chem.*, 2009, 1759–1766.
34 A. Bondi, *J. Phys. Chem.*, 1964, **68**, 441–451.
35 L. Falivene, Z. Cao, A. Petta, L. Serra, A. Poater, R. Oliva, V. Scarano and L. Cavallo, *Nat. Chem.*, 2019, **11**, 872–879.
